# Genotype–Phenotype Relations for the Dystonia-Parkinsonism Genes *GLB1*, *SLC6A3*, *SLC30A10*, *SLC39A14*, and *PLA2G6*: MDSGene Systematic Review

**DOI:** 10.3390/ijms26094074

**Published:** 2025-04-25

**Authors:** Jon Rodriguez-Antiguedad, Rajasumi Rajalingam, Clara Krüger, Daniel Teixeira-dos-Santos, Christine Sun, Elias Fernandez-Toledo, Alexia Duarte, Paula Saffie-Awad, Matthew J. Barrett, Joseph L. Flanigan, Maziar Emamikhah, Neepa Patel, Marta San Luciano, Christine Cooper, Natascha Bahr, Odinachi Oguh, Alissa Buhrmann, Merle Vater, Rabea Fuchshofen, Franca Vulinovic, Maik-Iven Parreidt, Anne Weissbach, Katja Lohmann, Christine Klein, Connie Marras, Sarah Camargos

**Affiliations:** 1 Movement Disorders Unit, Sant Pau Hospital, 08041 Barcelona, Spain; jrodriguezmu@santpau.cat; 2 Institut de Investigacions Biomèdiques-Sant Pau, 08041 Barcelona, Spain; 3 Medicine Department, Universitat Autònoma de Barcelona (UAB), 08193 Barcelona, Spain; 4 Centro de Investigación Biomédica en Red Enfermedades Neurodegenerativas (CIBERNED), 28031 Madrid, Spain; 5 Department of Psychiatry, Western Michigan University Homer Stryker MD School of Medicine, Kalamazoo, MI 49008, USA; sumi.rajalingam@gmail.com; 6 Morton and Gloria Shulman Movement Disorders Clinic and the Edmond J. Safra Program in Parkinson’s Disease, Toronto Western Hospital, University of Toronto, Toronto, ON M5T 2S8, Canada; christineeesun@gmail.com (C.S.); maziar.emamikhah2@gmail.com (M.E.); 7 Institute of Neurogenetics, University of Lübeck, 23562 Lübeck, Germany; krueger.clara@hotmail.de (C.K.); natascha.bahr@uni-luebeck.de (N.B.); alissa.buhrmann@student.uni-luebeck.de (A.B.); merlevater33@gmail.com (M.V.); r.fuchshofen@gmail.com (R.F.); maikiven.parreidt@student.uni-luebeck.de (M.-I.P.); katja.lohmann@uni-luebeck.de (K.L.); christine.klein@uni-luebeck.de (C.K.); 8 Department of Neurology, Hospital de Clinicas de Porto Alegre, Porto Alegre 90035-903, Brazil; danieltds1@gmail.com; 9 Facultad de Medicina, Pontificia Universidad Católica de Chile, Santiago 7820436, Chile; elias.mft@gmail.com; 10 Department of Internal Medicine, Health Sciences Sector, Federal University of Paraná, Curitiba 81531-980, Brazil; alexiaduarte@ufpr.br; 11 Clínica Santa María, Santiago 7520349, Chile; psaffie@gmail.com; 12 Department of Neurology, Virginia Commonwealth University, Richmond, VA 23220, USA; matthew.barrett@vcuhealth.org; 13 Department of Neurology, University of Virginia, Charlottesville, VA 22903, USA; jlf4m@uvahealth.org; 14 RUSH Parkinson’s Disease and Movement Disorders Program, Department of Neurological Sciences, RUSH University Medical Center, Chicago, IL 60612, USA; neepa_patel@rush.edu; 15 Department of Neurology, University of California San Francisco, San Francisco, CA 94143, USA; marta.sanlucianopalenzuela@ucsf.edu; 16 Department of Neurology, Medical University of South Carolina, Charleston, SC 29425, USA; cooperch@musc.edu; 17 Neurology Service, Ralph H. Johnson VA Medical Center, Charleston, CA 29401, USA; 18 Cleveland Clinic Luo Rico Center of Brain Health, Las Vegas, NV 89106, USA; odioguh@hotmail.com; 19 Center for Rare Diseases, University Clinic of Schleswig-Holstein, Campus Lübeck, 23538 Lübeck, Germany; anne.weissbach@uni-luebeck.de; 20 Institute of Systems Motor Science, Center of Brain, Behavior and Metabolism, University of Lübeck, 23562 Lübeck, Germany; 21 Movement Disorders Unit, Neurology Service, Internal Medicine Department, Hospital das Clínicas, The Federal University of Minas Gerais, Belo Horizonte 30130-100, Brazil

**Keywords:** dystonia, parkinsonism, *GLB1*, *SLC6A3*, *SLC30A10*, *SLC39A14*, *PLA2G6*, MDSGene

## Abstract

The Movement Disorders Society recommends the DYT/PARK prefix for genes where dystonia and parkinsonism are prominent in approximately half or more of patients. This systematic review explores the genotype–phenotype correlations of *GLB1*, *SLC6A3*, *SLC30A10*, *PLA2G6*, and *SLC39A14*—recently classified as DYT *SLC39A14* and historically linked to dystonia-parkinsonism. We searched PubMed and the Human Gene Mutation Database using standardized terms, including English-language, peer-reviewed publications up to February 2024. Following the MDSGene protocol, we extracted individual-level data on patients with biallelic pathogenic variants and at least one movement disorder. Features were marked “missing” if not explicitly reported. Of 1828 articles, 128 were eligible. We identified 386 patients and 262 variants. The median age at onset was 3 years for *GLB1*, 3 months for *SLC6A3*, 2.5 years for *SLC30A10*, 1.5 years for *SLC39A14*, and 16 years for *PLA2G6*. Missing data may reflect underreporting of negative findings. Case reports/serie, may bias toward atypical presentations. Our analysis showed dystonia-parkinsonism predominates in *SLC6A3* and *PLA2G6*, while *GLB1*, *SLC30A10*, and *SLC39A1* show predominantly dystonic phenotypes with a low frequency of parkinsonism. Ataxia was common in *GLB1* and *PLA2G6*. Awareness of these phenotypes is essential for early diagnosis and intervention, particularly in treatable conditions like *SLC30A10* or *SLC39A14*. The predominantly dystonic phenotype in *GLB1*, *SLC30A10*, and *SLC39A14* suggest that the DYT prefix may be more appropriate, highlighting the need to reconsider their nomenclature, and the importance of systematic reviews.

## 1. Introduction

Dystonia and parkinsonism are distinct syndromes with overlapping pathophysiology, primarily involving the cortico-basal ganglia-thalamic and cerebellar circuits, as well as the dopaminergic neurotransmission system [1]. Numerous structural, metabolic, drug-induced, autoimmune, and genetic disorders can simultaneously cause both syndromes (dystonia-parkinsonism) by disrupting these circuits. When no specific cause is identified after a thorough diagnostic workup, the syndrome is classified as idiopathic and, particularly in younger patients, a genetic origin is more likely [1]. Advances in molecular diagnostics have significantly broadened the genetic differential diagnosis.

The Movement Disorders Society (MDS) Task Force for the Nomenclature of Genetic Movement Disorders recommends the use of the DYT/PARK prefix for genes where dystonia and parkinsonism generally coexist or where both are prominent features in approximately half or more of the patients [2,3]. Additionally, their co-occurrence must be reported by at least two independent groups with sufficient evidence supporting pathogenicity. This prefix has been so far assigned to 12 conditions associated with pathogenic variants in the following genes: *TAF1*, *ATP1A3*, *GLB1*, *CP*, *SLC6A3*, *SLC30A10*, *PLA2G6*, *GCH1*, *TH*, *SPR*, *QDPR*, and *PTS* [2].

The first genetic condition associated with a dystonia-parkinsonism phenotype was described by Segawa in 1971 as a hereditary progressive dystonia with marked diurnal fluctuations. This condition, known as dopa-responsive dystonia (DRD), was later linked to pathogenic variants in *GCH1* [4]. Since then, several dopamine-responsive disorders sharing a common molecular basis that involves the dopamine synthesis pathway have been identified (*GCH1*, *TH*, *SPR*, *QDPR*, and *PTS*). These DYT/PARK genes have already been reviewed using MDSGene methodology [5]. Pathogenic variants in *ATP1A3* and *TAF1* also result in a DYT/PARK phenotype but have been or will be reviewed separately due to the phenotypic pleiotropy related to *ATP1A3* variants, and the X-linked inheritance pattern in individuals of Filipino ancestry with a specific disease-causing SVA insertion in *TAF1* [6]. Likewise, *CP*-associated disease will also be reviewed separately, as in the course of this study its primary phenotype was found to be ataxia.

This MDSGene systematic review complements the previous publications on DYT/PARK genes [5,6]. These genes encode proteins with diverse roles in cellular metabolism: *GLB1* encodes the lysosomal enzyme beta-galactosidase 1; *SLC6A3* encodes the dopamine transporter; *SLC30A10* encodes the Zn and Mn transporter type 10; and *PLA2G6* encodes the calcium-independent phospholipase A2 [7,8,9,10]. While pathogenic variants in *SLC39A14* (Mn transporter) were classically associated with dystonia-parkinsonism phenotypes, a recent update by the MDS Task Force for the Nomenclature of Genetic Movement Disorders classified this condition as DYT-*SLC39A14* [3,11]. Despite this new classification, we included *SLC39A14* in the current study to assess the appropriateness of this label based on a systematic review.

The main objective of this study is to provide a comprehensive overview of the phenotypic, demographic, and genotypic data of patients with potentially pathogenic variants in the aforementioned genes, allowing an assessment of the appropriateness of the current nomenclature, and highlighting key disease characteristics to improve clinical diagnosis and management.

## 2. Materials and Methods

### 2.1. MDSGene

MDSGene is an online knowledge base that provides a comprehensive and systematic overview of published data on movement disorder patients carrying pathogenic variants in movement disorder-linked genes [12]. Several systematic reviews from MDSGene have been published, offering clinicians detailed genotype and phenotype information, and also aiding in genetic counseling [5,13,14,15,16,17,18,19].

The systematic literature search and data extraction were conducted according to the standardized MDSGene protocol [12]. All data and detailed protocols are available on the MDSGene website (https://www.mdsgene.org (accessed on 28 February 2025)).

### 2.2. Literature Search

We conducted a systematic literature search for publications reporting individual-level data on patients with movement disorders associated with variants in *GLB1*, *SLC6A3*, *SLC30A10*, *SLC39A14*, and *PLA2G6*. The search was performed in the National Center for Biotechnology Information PubMed database (https://pubmed.ncbi.nlm.nih.gov/ (accessed on 28 February 2025)) using standardized search terms (Table S1). We included publications in English from peer-reviewed journals up to 12 February 2024. Additional potentially eligible studies were screened through relevant references within the included manuscripts, and through the Human Gene Mutation Database (HGMD). This systematic review adheres to the Preferred Reporting Items for Systematic Reviews and Meta-Analysis (PRISMA) guidelines, ensuring a transparent and comprehensive documentation of the review process.

### 2.3. Inclusion and Exclusion Criteria for Patients and Genetic Variants

Individuals were included for data abstraction if they were clinically affected by at least one movement disorder and had a confirmed biallelic variant in any of the five genes of interest (all with autosomal recessive inheritance).

Variants were included if they had a minor allele frequency (MAF) < 1% based on the highest MAF reported by ethnicity in the gnomAD database (https://gnomad.broadinstitute.org (accessed on 28 February 2025), version 4.1.0) and/or by screening in unaffected controls in the respective publication. We excluded variants that were classified as benign according to the MDSGene criteria (see Section 5).

### 2.4. Data Collection Process 

Data on demographics, genetics, clinical variables, and ancillary tests were extracted according to the standardized MDSGene protocol. The age at onset (AAO) variable refers specifically to the onset of the movement disorder. All the extracted variables are listed in Table S2. The percentages of each variable were calculated using all individuals in the denominator regardless of their missing status, assuming that the feature was not present. The missing data for the variables reported in this review for each gene are summarized in Table S3. The percentage of missing data is indicated in the text when it is deemed relevant for data interpretation.

For the genetic variants included from publications, additional genetic data (including chromosomal physical location, coding and genomic sequences, and protein information) were collected from databases such as VarSome (https://varsome.com (accessed on 28 February 2025)), MutationTaster (http://www.mutationtaster.org/ (accessed on 28 February 2025)), and Ensembl (https://www.ensembl.org/index.html (accessed on 28 February 2025)). All variants were mapped to the GRCh37/hg19 reference genome, and nomenclature was standardized based on the following transcripts: ENST00000307363/NM_000404 (*GLB1*), ENST00000270349/NM_001044 (*SLC6A3*), ENST00000366926/NM_018713 (*SLC30A10*), ENST00000359741/NM_015359 (*SLC39A14*), and ENST00000332509/NM_003560 (*PLA2G6*). Information from multiple publications referring to the same patient was compiled into a single entry.

### 2.5. Pathogenicity Scoring 

The degree of pathogenicity of a genetic variant was assessed as described in the MDSGene methods (https://www.mdsgene.org/methods (accessed on 28 February 2025)). In brief, a pathogenicity score for each variant is generated based on four criteria: co-segregation with the disease, frequency in the general population (http://gnomad.broadinstitute.org/ (accessed on 28 February 2025), version 4.1.0), the Combined Annotation Dependent Depletion (CADD, GRCh37-v1.7) score (https://cadd.gs.washington.edu/ (accessed on 28 February 2025)), and documented molecular evidence from in vivo and/or in vitro studies. Each criterion contributes points to a total score, which is used to classify variants as follows: benign (score < 5), possibly pathogenic (score 5–9), probably pathogenic (score 10–14), or definitely pathogenic (score > 14). This system has been adapted from the American College of Medical Genetics’ recommendations.

### 2.6. Statistical Analyses

Descriptive statistics were obtained for the demographic and clinical variables. Group differences were analyzed using the Mann–Whitney *U* and Kruskal–Wallis tests for the continuous variables and the *χ*^2^ test for the categorical variables. Continuous variables are reported as medians and interquartile ranges (IQR), while categorical variables are presented as counts and percentages. The association between the presence of a variant affecting a specific protein domain with the movement disorders phenotype was analyzed using the *χ*^2^ test. All analyses were performed using IBM SPSS software (v26). A two-tailed *p* value of < 0.05 was considered statistically significant for all tests.

## 3. Results

The PubMed literature search yielded 1868 citations, of which 128 met the criteria for data abstraction: 21 for *GLB1*, 11 for *SLC6A3*, 15 for *SLC30A10*, 10 for *SLC39A14*, and 71 for *PLA2G6* (Figure S1). A complete list of the articles included can be found in Table S4. As already mentioned in the introduction, *CP* will be reviewed separately, as the systematic review identified ataxia as its primary phenotype.

**Figure 1:**
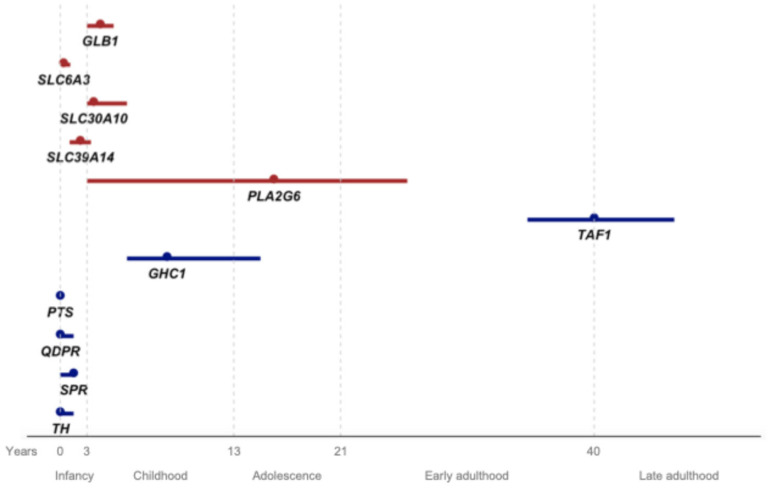
Age at onset for each gene from the present review (red) and previously reviewed DYT/PARK genes by Weissbach et al. and by Pauly et al., represented by the median (dot) and interquartile range (line). The x-axis represents age in years, with life stages assigned to each interval.

**Figure 2:**
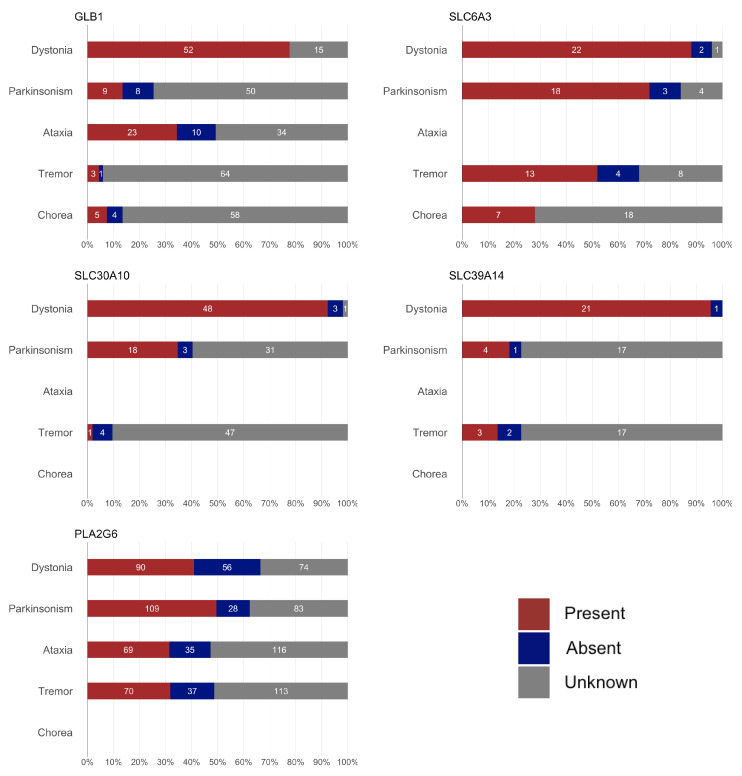
Summary of the frequency of movement disorders for each gene. The symptoms for which no bars are shown were not collected during data abstraction for the corresponding gene.

**Figure 3:**
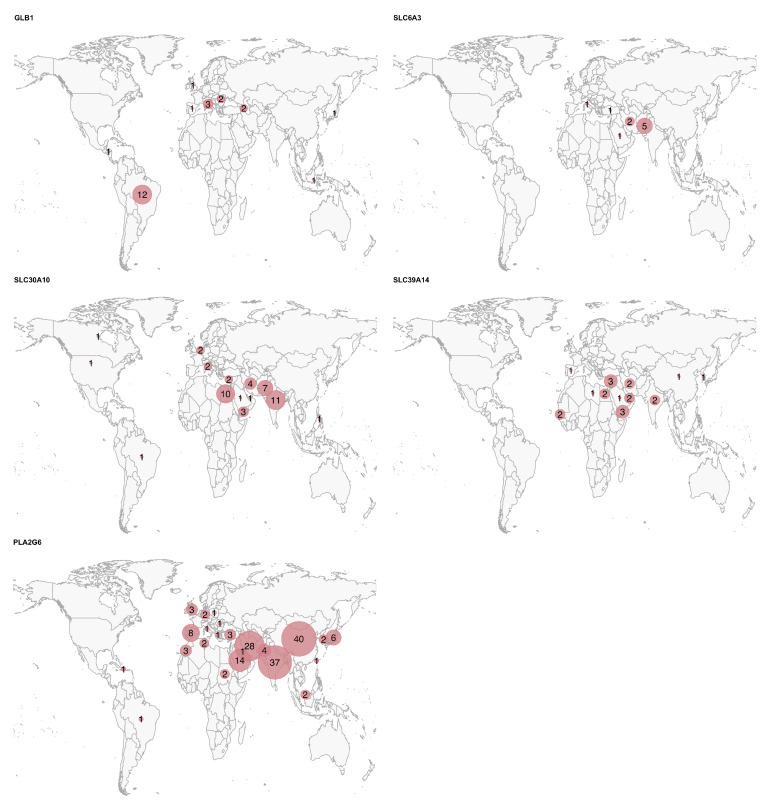
Geographic distribution of the patients for each gene. The circle size represents the relative number of cases per country for each disease, scaled independently within each map.

We included a total of 386 potentially pathogenic variant carriers, encompassing 262 distinct variants (Table 1 and Figure S2). The clinical and demographic data for all patients are summarized in Table 2, Figure 1 and Figure 2. The geographic distribution of the included patients is shown in Figure 3.

### 3.1. GLB1

We identified 67 *GLB1* potentially pathogenic variant carriers from 60 different families (10% consanguineous). Of these, 77.6% were compound heterozygotes, and 22.4% were homozygotes. A total of 58 different variants were identified, the majority of which were missense (81.4%).

The median AAO was 3 years (IQR 2–4) (Figure 1), while the median age at diagnosis was 7.1 years (IQR 2.7–14). The median age at examination (AAE) was 15 years (IQR 8.4–20.4). The most commonly reported initial symptoms were developmental delay or regression (17.9%), dysarthria (17.9%), and gait difficulties (14.9%).

Throughout the disease course, dystonia was observed in 77.6% of individuals (23.4% missing data). Craniofacial involvement occurred in 40.4%, while generalized dystonia was noted in 17.9%. Ataxia (40.4%) and parkinsonism (13.4%) were much less frequent. A dystonia-ataxia phenotype was identified in 21 individuals, whereas a dystonia-parkinsonism phenotype was present in only 9. The median AAO for individuals developing parkinsonism (12 years, IQR 4.3–19) was higher (*p* = 0.006) than for those developing dystonia (3 years, IQR 2–5) or ataxia (2 years, IQR 1.8–4). We found no significant differences in the prevalence of the main phenotypes (dystonia, parkinsonism, and/or ataxia) between patients with pathogenic variants inside or outside the glycosyl hydrolase domain (*p* = 0.350).

Other neurological manifestations included developmental disorders (49.3%), dysarthria (47.8%), upper motor neuron symptoms (44.8%), dysphagia (43.2%), hypotonia (34.3%), seizures (25.4%), and cognitive impairment (20.9%). The median AAO among individuals presenting with upper motor neuron symptoms, hypotonia, and seizures, was 2 years (IQR 1.2–3), 1.5 years (IQR 0.5–2), and 1.6 years (IQR 1–2.3), respectively.

Skeletal abnormalities, including spinal deformities, bone dysplasia, kyphoscoliosis, and dysostosis multiplex, were reported in 74.6% of cases. Short stature and dysmorphic features were noted in 37.3% and 16.4%, respectively. Less commonly reported features included ophthalmologic abnormalities, such as a red macula spot or corneal opacity (16.4%), hepatosplenomegaly (9%), and microcephaly (6%).

Magnetic resonance imaging (MRI) was abnormal in 18 out of 19 individuals with available data. Although specific abnormalities were rarely reported, the most common findings were cortical and/or basal ganglia atrophy (38.9%), putaminal and pallidal T2 hypointensity (22.2%), and posterior putaminal T2 hyperintensity (16.7%). The *wishbone* sign was reported in a single patient [20].

Data on treatment were very limited. A trial of miglustat was reported in four patients, with 75% showing a positive response, including improvements in gait and speech. Other treatments showed more variable positive responses: 1/1 patient with botulinum toxin, 1/1 with oral baclofen, 1/2 with dopaminergic drugs, and 0/2 with anticholinergics.

### 3.2. SLC6A3

We identified 25 *SLC6A3* potentially pathogenic variant carriers from 21 different families (61.9% consanguineous). Most affected individuals were homozygous (80%), with five compound heterozygous (20%). Among the 26 different variants, missense was the most prevalent (53.8%).

The median AAO was 3 months (IQR 3–9 months), the median AAE was 6 years (IQR 3.3–15.5), and the median age at diagnosis was 3 years (IQR 1.4–26.5). The most frequently reported initial signs and symptoms were dystonia (44%), hypotonia (32%), irritability and feeding problems (32%), and parkinsonism (24%). No statistically significant differences were found in the median AAO between the patients with and without parkinsonism (*p* = 0.914).

Dystonia was the most frequent movement disorder (88%) followed by parkinsonism (72%). Dystonia was generalized in 86.4%, and oromandibular involvement was present in 36.4%. Status dystonicus episodes were documented in 10 patients (45.5%). Rest tremor was present in 50% of the patients with parkinsonism. Dystonia and parkinsonism co-occurred in 64% and only one patient with atypical late-onset presentation did not develop either dystonia or parkinsonism [9]. We did not find differences in the prevalence of dystonia and/or parkinsonism between the patients with pathogenic variants inside or outside the transmembrane region (*p* = 0.100).

Developmental delay or regression was present in all patients except one with late-onset presentation (96%). Other associated neurological symptoms included spontaneous dyskinesia in 56%, hypotonia in 52%, eye movement disorders (saccade initiation failure, slow saccades, ocular flutter, and oculogyric crisis) in 44%, upper motor neuron signs in 40%, and dysarthria/anarthria in 36%. Orolingual dyskinesia was reported in nine patients (36%). Cognitive impairment was only reported in two patients.

Four patients with non-infantile atypical presentation were identified. Among these, three Pakistani siblings presented at age 11 with hand and head tremor [9]. Another individual presented at age 28 with hand tremor, which progressed to a universal tremor in all extremities accompanied by parkinsonism [21].

The CSF homovanillic acid/5-hydroxyindoleacetic acid (HVA:5-HIAA) ratio was elevated (median 9.5, IQR 6.8–12.1) in all individuals with available data, indicating an increased dopamine-to-serotonin metabolite ratio.

Dopaminergic drugs were administered in 76% of individuals, with minimal or no response in 57.9%. Other treatments, such as deep brain stimulation, anticholinergic drugs, baclofen, benzodiazepines, VMAT2 inhibitors, carbamazepine, selegiline, and amantadine were tried in a few patients with minimal or no improvement in most cases.

### 3.3. SLC30A10

We identified 52 potentially pathogenic variant carriers from 35 families (80% consanguineous). The most common ethnicity was Indian (25%). All individuals were homozygous. Among the 27 identified variants, missense (33.3%) and frameshift (25.9%) were the most frequent types.

The median AAO was 2.5 years (IQR 2–5). The median AAE was 10 years (IQR 4.5–15.5). The most common initial symptoms were dystonia (61.5%) and gait difficulties (34.6%) (missing data 25%). Two siblings with a homozygous c.1235delA variant, affecting the region for plasma membrane localization, had onset at ages 47 and 57, developing a dopamine-resistant parkinsonian syndrome with freezing of gait and without dystonia [22].

Dystonia was the predominant movement disorder (92.3%), while parkinsonism occurred at a considerably lower frequency (34.6%). Onset of dystonia was reported in the lower limbs in 27.1% and progressed to generalized dystonia in 41.7%. Co-occurrence of dystonia and parkinsonism was observed in 17 patients (32.7%). No differences were found in AAO between the patients with and without parkinsonism (*p* = 0.260). We did not find differences in the prevalence of dystonia and/or parkinsonism between the patients with pathogenic variants inside or outside the transmembrane region or the region for plasma membrane localization (*p* = 0.150).

Dysarthria/anarthria was the only additional neurological sign in a significant percentage of patients (46.2%). A characteristic *cock walk* was reported in three patients. Cognitive impairment (11.5%) and developmental disorders (11.5%) were infrequent.

T1 hyperintensities of the basal ganglia (90.4%), hypermanganesemia (94.2%), and polycythemia (94.2%) were nearly universal findings. Reduced ferritin (30.8%) and hepatomegaly or cirrhosis (23.1%) were also common findings in ancillary tests.

Treatment trials with the Mn chelator disodium calcium edetate were reported in 18 patients and with dopaminergic drugs in 16 patients, 12 of whom had parkinsonism. A positive response was observed in 88.9% of the patients treated with disodium calcium edetate and in 31.3% of those treated with dopaminergic drugs. An ethylenediaminetetraacetic acid (EDTA) trial was also conducted in five patients, with a positive response in four of them, while penicillamine treatment showed a positive response in only 1/5 trials.

### 3.4. SLC39A14

We identified 22 *SLC39A14* potentially pathogenic variant carriers from 18 different families (72.2% consanguineous). Although several nationalities were represented, 54.5% of the patients were originally from the Middle East. All affected individuals were homozygous except for one compound heterozygous patient who otherwise had a typical disease course. In total, we identified 15 different variants, with missense variants being the most frequent (60%).

The median AAO was 1.5 years (IQR 0.7–2.3). The median AAE was 5 years (IQR 2–10). The most frequently reported initial symptoms were dystonia (54.5%), developmental delay/regression (50%), and gait/balance disorders (22.7%). One homozygous individual (c.1066G > A) had a late onset at the age of 18 years, presenting with dysarthria and generalized dystonia, and exhibited a benign course with stable symptoms.

Dystonia was the most frequently reported symptom (95.5%). The onset was in lower limbs in 27.3% and progressed to generalized dystonia in 59.1%. Parkinsonism was reported in only four patients, all of whom exhibited a DYT/PARK phenotype. Developmental disorder was the second most frequently reported symptom (59.1%). The only patient without dystonia presented with developmental delay, spasticity, and hyperreflexia as primary symptoms. We did not find differences in the prevalence of dystonia and/or parkinsonism between patients with variants inside or outside the ZIP Zn transporter region (*p* = 0.130).

Other frequently associated symptoms included spasticity (50%), dysarthria (45.5%), hyperreflexia (45.5%), tip-toe gait (27.3%), and microcephalus (27.3%). Cognitive impairment (18.2%) and neuropsychiatric symptoms (4.5%) were rare.

Hypermanganesemia and basal ganglia T1 hyperintensities were present in all patients who had Mn blood levels or MRI data available (90.9%). Seven patients (31.8%) showed pituitary gland hyperintensity on T1 MRI. One patient (4.5%) had low ferritin levels, and none of the patients exhibited polycythemia (0%).

A predominantly positive response to treatment was observed only with dissodium calcium edetate (7/10) and intrathecal baclofen (2/2). A clearly positive response with other therapies was less common: anticholinergics (3/7), dopaminergic treatment (0/7), and benzodiazepines (1/7).

### 3.5. PLA2G6

We identified 220 *PLA2G6* potentially pathogenic variant carriers from 183 distinct families (45.9% consanguineous). The most common countries of origin were China (18.2%), India (18.2%), and Iran (12.7%). Among the 136 identified variants, the most frequent were c.991G > T (25%) and c.2222G > A (21.3%), both missense variants. Missense variants were predominant (73.5%). Of the individuals, 117 were homozygous, and 103 compound heterozygous.

The median AAO was 16 years (IQR 2–26), with 39.1% presenting in infancy-childhood and 36.4% presenting in early-adulthood (missing data 14.5%). The median AAE was 20 years (IQR 7–31). The most frequently reported initial symptoms were developmental disorders (16.8%), gait disturbances (15%), parkinsonism (12.3%), and psychiatric symptoms (8.6%). Notably, c.991C > T (median AAO 30 years, IQR 23.5–32.5) and c.2222G > A (median AAO 18 years, IQR 10–23) had a later onset compared to other rarer variants (median AAO 5.5 years, IQR 1.5–23) (*p* < 0.001). Interestingly, two Sudanese patients presented with late-onset Parkinson’s disease in their sixties, without iron deposition on MRI, and with genetic analysis revealing compound heterozygosity for c.2071_2073delGTC and c.956C > T (both classified as possibly pathogenic).

Parkinsonism (49.5%), dystonia (40.9%), and ataxia (31.4%) were the most common movement disorders. Among the patients with parkinsonism, resting tremor was observed in 33.9%, 44% developed dyskinesias, and 13.8% experienced motor fluctuations. In patients with dystonia, 22.2% had generalized dystonia, and craniofacial involvement was present in 15 patients (16.7%). Overall, 55 (25%) individuals exhibited a DYT/PARK phenotype, and 144 (65.5%) had either dystonia or parkinsonism. Compared to early-adulthood onset, cases with infantile-childhood onset had a higher proportion of ataxia (42.3% vs. 21.5%, *p* = 0.004), a lower proportion of parkinsonism (14.4% vs. 92.4%, *p* < 0.001), and a similar proportion of dystonia (39.4% vs. 44.3%, *p* = 0.802). Compared to other variants, the patients with c.991G > T (82.9% vs. 58%, *p* = 0.006) and c.2222G > A (85.7% vs. 58%, *p* = 0.005) were more likely to manifest dystonic and/or parkinsonian phenotypes. However, taking all the variants together, we found no association in the prevalence of the main phenotypes (dystonia, parkinsonism, and/or ataxia) between the patients with variants inside or outside the transmembrane or the ankyrin repeat regions (*p* = 0.310).

Other neurological features included cognitive impairment (57.3%), upper motor neuron symptoms (55.9%), ocular findings, such as nystagmus, strabismus, optic atrophy, and oculomotor abnormalities (40.9%), developmental disorders (35%), neuropsychiatric symptoms (25%), dysarthria or anarthria (24.1%), seizures (21.4%), and hypotonia (19.5%).

MRI abnormalities were reported in 91.9% of the individuals with available results (84.1%, of whom 94 patients had an infantile-childhood onset and 65 an early-adulthood onset). The most common findings included cerebellar atrophy (75.7%), pallidal and/or substantia nigra T2 hypointensities (40%), cerebral atrophy (26.5%), T2 cerebellar hyperintensities (10.8%), claval hypertrophy (10.8%), and white matter hyperintensities (8.1%). Presynaptic dopaminergic terminal testing was abnormal in 36 out of 48 patients.

Among the 103 trials with dopaminergic drugs or amantadine, 77.7% exhibited a positive or transient response, particularly with regard to parkinsonian symptoms. The response to DBS was favorable in all individuals who underwent the procedure (7/7). In contrast, anticholinergic and other drugs were used less frequently and demonstrated more variable efficacy.

**Table 2:** Summary of demographic and clinical data for each gene.

	*GLB1*	*SLC6A3*	*SLC30A10*	*SLC39A14*	*PLA2G6*
**n**	67	25	52	22	220
**Families (consang.)** ^†^	60 (6)	21 (13)	35 (28)	18 (13)	183 (84)
**Sex, female**	40.3%	52%	42.3%	68.2%	46.4%
**AAO, years** ^‡^	3 (2–4)	0.3 (0.3–0.8)	2.5 (2–5)	1.5 (0.7–2.3)	16 (2–26)
**AAD, years** ^‡^	7.1 (2.7–14)	3 (1.4–26.5)	12 (9–23)	4 (1.2–8)	23 (8–32)
**Initial symptoms**	Developmental disorder 12/47 Dysarthria 12/47 Gait difficulties 10/47	**Dystonia** 11/19 Hypotonia 8/19 Irritability/feeding problems 8/19 Parkinsonism 6/19	**Dystonia** 32/39 Gait difficulties 18/39	**Dystonia** 12/20 **Developmental disorder** 11/20 Gait difficulties 5/20	Developmental disorder 37/115 Gait difficulties 33/115 Parkinsonism or bradykinesia 27/115
**Movement disorders**	**Dystonia** 52/52 Ataxia 23/33 Parkinsonism 9/17	**Dystonia** 22/24 **Parkinsonism** 18/21 **Any dyskinesia** 14/18 **Orolingual dyskinesia** 9/9	**Dystonia** 48/51 Parkinsonism 18/21	**Dystonia** 21/22 Parkinsonism 4/5	**Parkinsonism** 109/137 **Dystonia** 90/146 Ataxia 69/104
**Combination of movement disorders**	Dystonia-ataxia 21/31 Dystonia-parkinsonism 9/17	**Dystonia-parkinsonism** 16/20	Dystonia-parkinsonism 15/21	Dystonia-parkinsonism 4/5	Dystonia-parkinsonism 55/101 Dystonia-ataxia 32/87 Parkinsonism-ataxia 28/92
**Other neurological symptoms**	**Developmental disorder** 33/42 **Dys/anarthria** 32/32 **Upper motor neuron** 30/36 **Dysphagia** 29/42 Hypotonia 23/33 Seizures 17/44 Cognitive impairment 14/22 Ophthalmologic abnormal 11/43	**Developmental disorder** 24/25 **Hypotonia** 13/14 **Eye movements abnormal** 11/13 **Upper motor neuron** 10/10 **Dys/anarthria** 9/12	**Dys/anarthria** 24/25 *Cock-walk* gait 3/3	**Developmental disorder** 13/15 **Spasticity** 11/11 **Hyperreflexia** 10/11 **Dys/anarthria** 10/10 Tip-toe gait 6/6 Cognitive impairment 4/10	**Cognitive impairment** 126/159 **Upper motor neuron** 123/145 **Ocular abnormal** 90/119 **Developmental disorder** 77/123 Neuropsychiatric 55/87 Dys/anarthria 53/73 Seizures 47/115
**Non-neurological symptoms**	**Skeletal abnormalities** 50/55 **Short stature** 25/37 Hepatosplenomegaly 6/50	-	Cirrhosis/hepatomegaly 12/27	Microcephaly 6/8	-
**Laboratory test abnormalities**	-	**Increased CSF HVA:5-HIAA** 17/17	**Hypermanganesemia** 49/51 **Polycythemia** 49/51 Low ferritin 16/29	**Hypermanganesemia** 20/20 Low ferritin 1/5 Polycythemia 0/15	-
**MRI abnormalities**	Atrophy, cortical and/or basal ganglia 7/18 T2 hypointensity, basal ganglia 4/18 T2 hyperintensity, posterior putaminal 3/18 Wishbone sign 1/18	-	**T1 hyperintensity, basal ganglia** 47/48	**T1 hyperintensity, basal ganglia** 20/20 T1 hyperintensity, pituitary gland 7/20	**Atrophy, cerebellar 140/185** T2 hypointensity, basal ganglia 74/185 Atrophy, cerebral 59/185 T2 hyperintensity, cerebellar 20/185 Claval hypertrophy 20/185 T2 hyperintensity, white matter 15/185
**Treatment, positive response**	Miglustat 3/4	Dopaminergic drugs 8/19	Disodium calcium edetate 16/18 Dopaminergic drugs 5/16	Dissodium calcium edetate 7/10 Anticholinergics 3/7 Intrathecal baclofen 2/2 Benzodiazepines 1/7 Dopaminergic drugs 0/7	Dopaminergic drugs 72/90 Amantadine 8/13 DBS 7/7 Baclofen 2/4
**Suggested prefix**	DYT	DYT/PARK	DYT	DYT	DYT/PARK

^†^ Number of families (number of consanguineous families); ^‡^ AAO and AAD are presented as median (IQR). Variables are expressed as a ratio: variable present/total of cases with the variable recorded (excluding missing values). Variables where prevalence exceeds 35% of the total n (including missing values) are highlighted in bold. AAO: age at onset; AAD: age at diagnosis; MRI: magnetic resonance imaging; HVA: homovanillic acid; HIAA: 5-hydroxyindoleacetic acid; DBS: deep brain stimulation.

## 4. Discussion

This review on *GLB1*, *SLC6A3*, *SLC30A10*, *SLC39A14*, and *PLA2G6* is the third MDSGene review series on DYT/PARK genes, following previous articles on DRD genes and X-linked dystonia-parkinsonism [5,6]. It aims to complement those reviews as well as future publications on *CP* and *ATP1A3*, which will be analyzed separately for their unique features. With the exception of *PLA2G6*-associated neurodegeneration (PLAN), which presents with a DYT/PARK phenotype later in life, these conditions represent neurological disorders with a typical infancy-to-childhood onset, characterized by dystonia associated with other movement disorders in varying proportions. However, we observed that the proportion of individuals manifesting parkinsonism in *GLB1*, *SLC30A10*, and *SLC39A14* is lower than expected under the DYT/PARK nomenclature, where dystonia and parkinsonism should generally coexist or be prominent features in approximately half or more of the patients. This finding calls for reconsideration in the MDSGene nomenclature for these genes and highlights the importance of such systematic reviews.

Biallelic variants causing a loss of function in *GLB1* result in altered beta-galactosidase activity. This dysfunction leads to two distinct lysosomal storage diseases, depending on whether the variant impairs the catalytic degradation of gangliosides or the degradation of keratan sulfate-bound oligosaccharides: GM1 gangliosidosis, a primary neurological disease, and mucopolysaccharidosis type IVB, a primary skeletal disease. GM1 gangliosidosis is a spectrum typically categorized into three subtypes based on the AAO: type I (infantile), type II (juvenile), and type III (adulthood) [23]. An association has been suggested between severe infantile forms and variants affecting the core or active enzyme regions of the 3D protein structure, as well as between later onset forms and variants affecting the surface of beta-galactosidase [24]. Dystonia, often generalized with characteristic facial involvement, is the most common movement disorder [25]. It may be accompanied by ataxia, usually seen at younger ages, or parkinsonism, more common in patients with a later onset [25]. Despite this general age distribution, any movement disorder can occur at any age. Although GM1 gangliosidosis is classified as DYT/PARK-*GLB1* due to the type III phenotype, this disease represents a continuum where dystonia is the clearly predominant symptom, often accompanied by other movement disorders [2]. Therefore, based on this review, the prefix DYT-*GLB1* should be considered for this condition. Despite a few trials with miglustat, a drug aimed at reducing sphingolipid production, current therapies for GM1 gangliosidosis are primarily symptomatic [10].

The data regarding pathogenic variants in *SLC6A3* are consistent with the previous literature [26]. The classical disease has an onset within the first year of life, often presenting with nonspecific symptoms, such as hypotonia or feeding difficulties, or with movement disorders in the form of dystonia or parkinsonism. Throughout its progression, developmental delay or regression, dystonia, and/or parkinsonism are almost universal, making the DYT/PARK prefix appropriate for this gene. Certain features, such as spontaneous orofacial dyskinesias, oculogyric crises, and an elevated CSF HVA:5-HIAA ratio, are characteristic and should guide diagnosis. As more cases are reported, the spectrum of the disease is expanding to include adolescent-onset cases, neuropsychiatric-predominant cases, and autosomal dominant-negative variants [26]. Treatment is still symptomatic and, despite its phenotypic similarities to DRDs, the dopaminergic response is more erratic [26].

SLC30A10 pathogenic variants are associated with altered Mn transport across biological membranes, leading to its accumulation in the brain and liver [8]. This condition, known as hypermanganesemia with dystonia 1, typically presents in infancy with generalized or multifocal dystonia, most often beginning in the lower limbs. This results in gait abnormalities, with a distinctive cock-walk gait pattern reported in some patients. Although previously classified as a DYT/PARK condition, according to this review, the number of patients with parkinsonism is low, while dystonia is present in nearly all patients, making DYT-SLC30A10 a more appropriate designation [2]. Chelation therapies, such as disodium calcium edetate, are usually effective in alleviating symptoms and stabilizing Mn levels, and are considered the cornerstone of the treatment [8].

*SLC39A14*-deficiency, also known as hypermanganesemia with dystonia 2, results from biallelic variants causing loss of function of a transporter of Zn, Mn, Fe, and Cd [11]. This malfunction leads to excessive Mn absorption and its accumulation primarily in basal ganglia. Despite fewer than 50 reported cases, the clinical phenotype in the literature is relatively consistent. Most patients develop infantile-onset progressive isolated dystonia, often accompanied by developmental delay, gait abnormalities, and dysarthria. The original publication on *SLC39A14* reported patients with concurrent dystonia and parkinsonism, initially leading to its classification as a progressive parkinsonism-dystonia syndrome [11]. However, subsequent case reports, including those reviewed in our study, have shown that, similar to *SLC30A10*, parkinsonism is a much less common symptom. This finding aligns with the recent recommendations from the MDS Genetic Nomenclature Task Force, which reclassified *SLC39A14*-deficiency as a DYT phenotype (DYT-*SLC39A14*) [3]. The absence of polycythemia and the rarity of cognitive or psychiatric symptoms help differentiate this disease from *SLC30A10*-related hypermanganesemia and acquired hypermanganesemia, respectively [27]. Dystonia is typically pharmacorresistant and besides disodium calcium edetate, which can slow disease progression, and intrathecal baclofen, symptomatic treatments are usually ineffective [11].

PLAN displays a bimodal distribution with distinct phenotypes based on the AAO. The earliest-onset phenotype is named infantile neuroaxonal dystrophy (INAD), characterized by psychomotor regression, hypotonia, and spastic paraparesis, and typically presents in infancy, while atypical neuroaxonal dystrophy (ANAD) manifests during childhood with a more variable presentation, often including ataxia, developmental delay, psychiatric, and ocular-visual symptoms [7]. In late-adolescence and early-adulthood onset, patients usually develop a dystonia-parkinsonism or early-onset PD phenotype, which are generally responsive to dopaminergic therapy [7]. Notably, the two common variants c.991C > T and c.2222G > A, associated with a later onset of disease, suggest distinct genotype–phenotype correlations and highlight the potential for selective brain vulnerability depending on the specific variant involved.

Experimental models are essential for deepening the understanding of disease mechanisms. For instance, the GLB1 knockout mouse model, which recapitulates the natural history of type II GM1 gangliosidosis, has proven valuable for exploring potential therapeutic strategies [28]. Additionally, recent findings suggest that GLP-1 receptor agonists may ameliorate neurodegeneration in murine models of infantile neuroaxonal dystrophy linked to *PLA2G6* [29].

In Figure 1, we present the AAO for the conditions associated with the genes reviewed in this article, as well as those previously reviewed by MDSGene (*TAF1*, *GHC1*, *PTS*, *QDPR*, *SPR*, and *TH*) [5,6]. Some of these latter genes (*PTS*, *QDPR*, *SPR*, and *TH*) are linked to childhood-onset diseases, overlapping with the AAO of the genes discussed in the present study, particularly *SLC6A3*. While certain features like seizures or oculogyric crisis may be found in *GLB1* and *PTS*, or *SLC6A3* and *SPR*, respectively, levodopa response is more inconsistent in *GLB1*, *SLC6A3*, *SLC30A10*, and *SLC39A14*, and should guide the diagnosis. The childhood onset of *GHC1*-associated disease may overlap with PLAN; however, when PLAN manifests before adolescence, it is typically accompanied by a much more severe phenotype with additional neurological symptoms. Lastly, *TAF1* has a distinct AAO, unique demographic characteristics (Filipino ancestry), and genetic features (X-linked), clearly distinguishing it from other diseases.

Finally, for some of these genes, a significantly higher prevalence (or reporting rate) of the disease was observed in specific countries: China, India, and Iran for *PLA2G6*; India and Pakistan for *SLC30A10*; and Middle Eastern countries, such as Egypt, Yemen, Iran, Saudi Arabia, and the United Arab Emirates for *SLC39A14*. The high rates of recessive diseases in these regions are likely attributable to cultural practices of consanguinity and endogamy in the Greater Middle East, North Africa, and Central Asia, combined with the tendency for large family sizes [30].

This study is not without limitations. The most evident limitation is the high proportion of missing data. Many patient data were only available in summary tables, with no detailed descriptions in the text. However, we classified a feature as “missing” (rather than “absent”) if it was not explicitly mentioned in the text. While this approach aims to avoid making assumptions, in many instances missing features might be absent due to word count restrictions or a tendency not to report negative findings. Given the predominantly neurological presentation of these conditions, it is likely that missing data on cardinal signs/symptoms, such as parkinsonism in *GLB1*, *SLC30A10*, and *SLC39A14*, reflect their actual absence. Nevertheless, we cannot exclude the possibility that subtle signs/symptoms may have been overlooked. Another important limitation is that the clinical data were derived from published individual case reports/series, which may introduce bias toward more atypical phenotypes. While GM1 gangliosidosis and PLAN are relatively more common diseases, the total number of reported cases in the literature for *SLC6A3*, *SLC30A10*, and *SLC39A14* is approximately 50 for each condition. Consequently, the data in this review for these latter genes seem more reliable, as they include a higher percentage of the total cases. We acknowledge the potential over- or underrepresentation of certain geopolitical regions, as shown in Figure 3. This imbalance may reflect the availability of published data, reporting practices, or access to genetic testing in different parts of the world. As such, caution is warranted when generalizing findings to underrepresented populations. In any case, the interpretation of the data should take these important limitations into account. By delineating gene-specific phenotypic patterns, awareness of early-onset dystonia with hypermanganesemia, and identification of characteristic MRI or CSF features associated with specific genes, clinicians can better prioritize differential diagnoses, initiate early interventions in treatable cases, and ensure appropriate referral for genetic counseling. These strategies can guide more targeted screening and improve diagnostic accuracy worldwide, especially in underserved regions.

## 5. Conclusions

This review highlights the similarities and differences among five neurological conditions typically presenting with early-onset dystonia and/or parkinsonism. With the exception of *SLC6A3* and *PLA2G6*, which primarily manifest with dystonia and parkinsonism as key features, we found that *GLB1*, *SLC30A10*, and *SLC39A14* predominantly exhibit a dystonic phenotype, sometimes accompanied by other movement disorders, such as parkinsonism or ataxia. A review of the nomenclature by the MDS Genetic Nomenclature Task Force should be undertaken given these findings. Increasing awareness of the phenotypes associated with these rare conditions is essential not only for guiding the selection and interpretation of genetic and complementary tests, but also for enabling early intervention, particularly in treatable conditions like *SLC30A10* or *SLC39A14* deficiency. Future reports will further refine and expand our understanding of the spectrum of these diseases. Although our review focuses on monogenic causes of dystonia-parkinsonism, we acknowledge that the phenotypic variability observed across individuals may be influenced by additional genetic or epigenetic factors. Recent advances in next-generation sequencing have revealed potential gene–gene interactions, digenic inheritance, and polygenic contributions that may modify disease expression. Moreover, epigenetic mechanisms—such as DNA methylation and chromatin remodeling—may further complicate the clinical presentation and progression of these disorders.

## Figures and Tables

**Table 1:** Summary of the genetic data for each gene.

	*GLB1*	*SLC6A3*	*SLC30A10*	*SLC39A14*	*PLA2G6*	Total
Genotype						
Homozygous	15	20	52	21	117	225
Compound heterozygous	52	5	0	1	103	161
Heterozygous	0	0	0	0	0	0
Total	67	25	52	22	220	386
Type of variant						
Missense	47	14	9	10	101	181
Nonsense	4	2	4	2	13	25
Structural variant	0	3	1	0	4	8
Frameshift	2	1	7	2	9	21
Splice site	4	4	1	1	7	17
In-frame indel	0	2	5	0	2	9
Silent	1	0	0	0	0	1
Total	58	26	27	15	136	262
Pathogenicity						
Definitely	16	5	1	0	3	25
Probably	39	19	25	15	109	207
Possible	3	2	1	0	24	30
Total	58	26	27	15	136	262

## Data Availability

All data and detailed protocols are available on the MDSGene website (https://www.mdsgene.org (accessed on 28 February 2025)).

## Supplementary Materials

The following supporting information can be downloaded at: https://www.mdpi.com/article/10.3390/ijms26094074/s1.
