# Supplementary material for: Genotype–Phenotype Relations for the Dystonia-Parkinsonism Genes GLB1, SLC6A3, SLC30A10, SLC39A14, and PLA2G6: MDSGene Systematic Review
Source: Int J Mol Sci. 2025 Apr 25;26(9):4074. doi: 10.3390/ijms26094074 (PMC12071818; doi:10.3390/ijms26094074)
Supplement: Supplementary file 1 [file ijms-26-04074-s001.zip › ijms-3568137-supplementary.pdf]

Supplementary materials

Figure S1. Flowchart of the study selection process

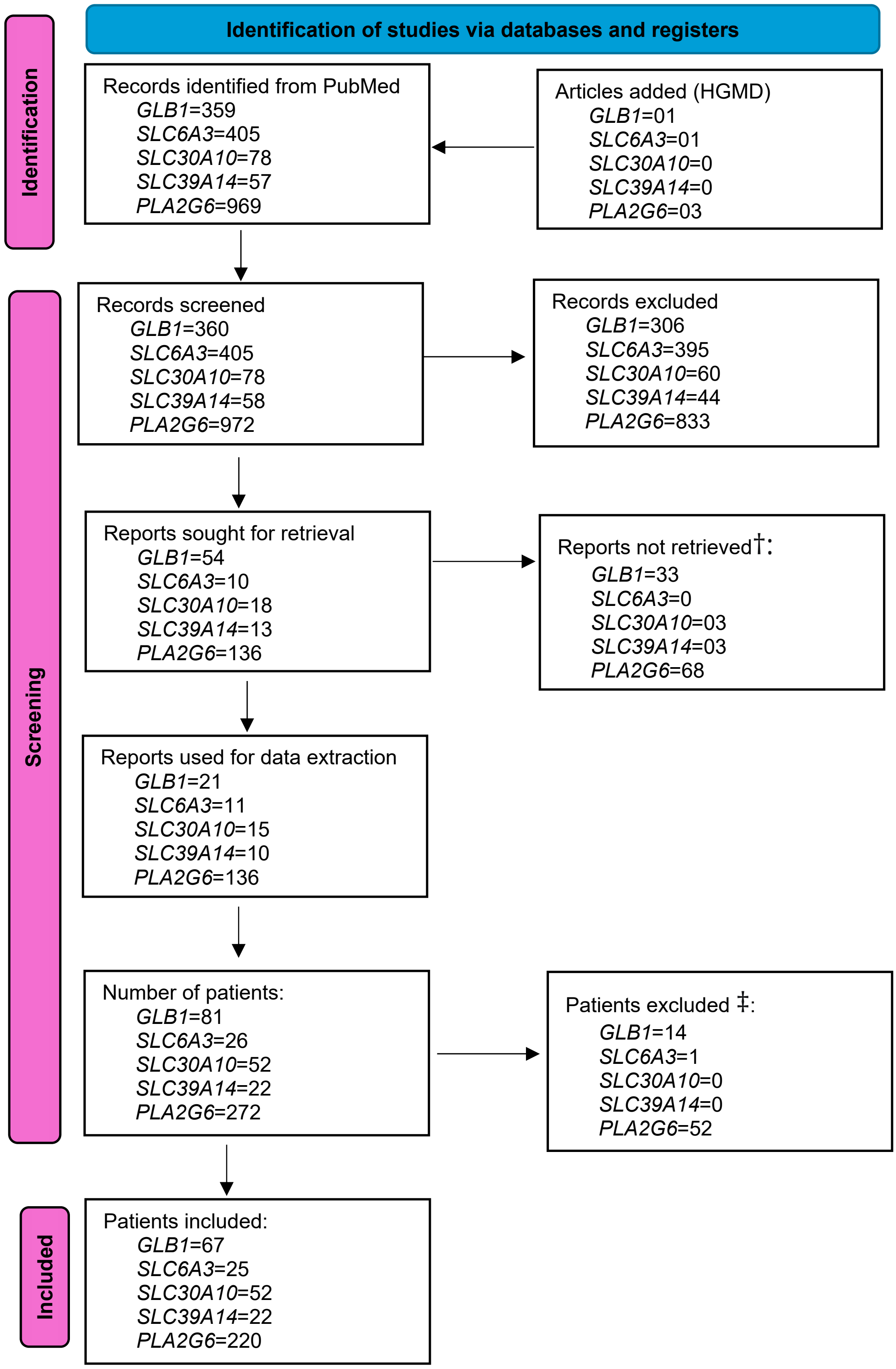

†No clinical or genetic data at the individual level, review paper, molecular genetic study without patients, Drosophila or zebrafish study, mouse model, protein expression, in vitro study.

‡No movement disorder phenotype, single heterozygous carriers, contradictory nomenclature, asymptomatic carrier, variant classified as benign or digenic

Figure S2. Schematic representation of *GLB1*, *SLC6A3*, *SLC30A10*, *SLC39A14*, and *PLA2G6* (A-E) (left) and the corresponding proteins (right). Exons are depicted as boxes, introns as lines, and untranslated regions in gray. Protein domains are highlighted in different colors

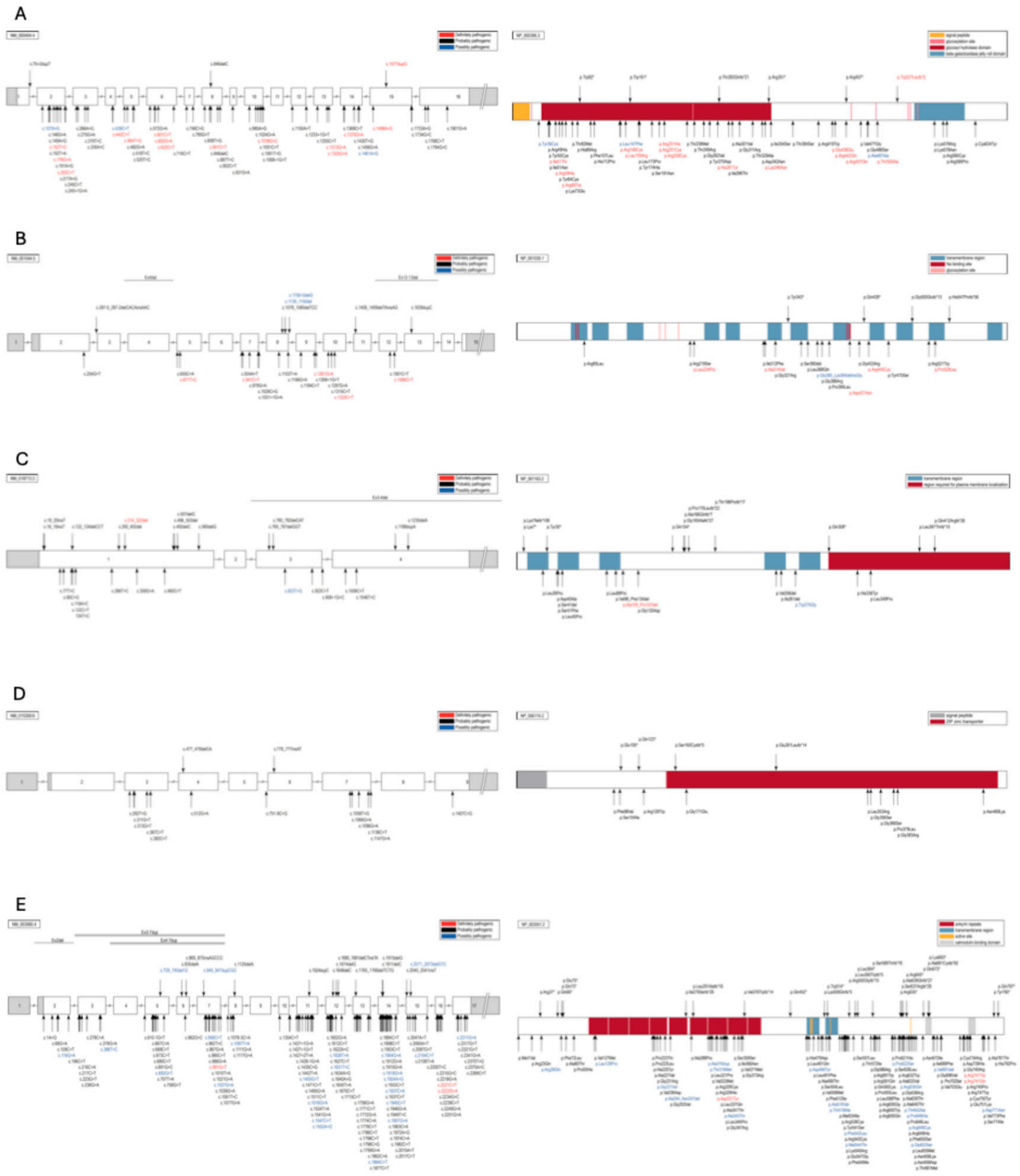

Table S1. Terms used in the Pubmed literature search for each gene

Gene	Search terms
GLB1	(dystoni* OR parkinson* OR GM1 gangliosidosis) AND (GLB1 OR beta galactosidase OR galactosidase beta 1 OR EBP OR ELNR1 OR MPS4B 3p22.3) AND (gene* OR genetic* OR mutation* OR mutated) AND “english”[Language]
SLC6A3	(dystoni* OR parkinson* OR ataxia OR cerebellar OR episodic ataxia OR paroxysmal ataxia OR channelopathy OR familial periodic ataxia OR Periodic vestibulocerebellar ataxia OR Acetazolamide responsive episodic ataxia OR hereditary spastic paraplegia OR paroxysmal movement OR tremor OR myoclonus OR chorea OR spastic paraplegia OR spastic paraparesis OR HSP) AND (SLC6A3 OR solute carrier family 6 member 3 OR PKDYS OR 5p15.33) AND (gene* OR genetic* OR mutation* OR mutated) AND “english”[Language]
SLC30A10	dystonia OR parkinson)* AND (SLC30A10 OR solute carrier family 30 member 10 OR ZNT8 OR ZRC1 OR HMDPC OR ZNT10 OR ZnT-10 OR HMNDYT1 OR 1q41) AND (gene* OR genetic* OR mutation* OR mutated) AND “english”[Language]
SLC39A14	(ataxia OR ataxic OR cerebellar OR channelopathy OR dystonia OR dystonic OR parkinson* OR paroxysmal movement OR tremor OR myoclon* OR chorea OR choreo* OR choreatic OR spastic paraplegia OR spastic paraparesis OR HSP OR Strümpell* OR hyperkinetic OR “movement disorder” OR dyskinesia OR dyskinetic OR hypermanganes*) AND (cig19 OR HCIN OR HMNDYT2 OR LZT-Hs4 OR NET34 OR ZIP14 OR KIAA0062 OR “Solute carrier family 39” OR “ZIP zinc transporter 4” OR “Zrt- and Irt-like protein 14” OR “Zrt-. Irt-like protein 14” OR 8p21.3 OR 8p21) AND "english"[Language]
PLA2G6	(ataxia OR ataxic OR cerebellar OR channelopathy OR dystonia OR dystonic OR parkinson* OR paroxysmal movement OR tremor OR myoclon* OR chorea OR choreo* OR choreatic OR spastic paraplegia OR spastic paraparesis OR HSP OR Strümpell* OR hyperkinetic OR “movement disorder” OR dyskinesia OR dyskinetic OR “brain iron accumulation”) AND (PLA2G6 OR “CaI-PLA2” OR “GVI” OR INAD1 OR iPLA2 OR “IPLA2-VIA” OR “iPLA2beta” OR NBIA2 OR NBIA2A OR NBIA2B OR PARK14 OR PLA2 OR PNPLA9 OR “Phospholipase a2” OR “Patatin-like phospholipase” OR 22q13.1) AND "english"[Language]

Table S2. List of all the extracted variables

**General information about the publication**
Pubmed ID
Author and year of publication
Study design
Genetic methods used to identify variants
**Patient characterization**
Individual patient ID
Family ID
Index patient
Sex
**Demographic information**
Ethnicity
Country of origin
**Clinical information**
General
Clinical Status (affected, unaffected, uncertain, unknown)
Consanguinity
Positive family history for the mutation associated phenotype
Age at examination in years
AAO in years
AAO classification (infancy, childhood, adolescence, early adulthood, late adulthood)
Disease duration in years
Age at clinical diagnosis in years
Age at death
Reported initial signs and symptoms
Motor signs and symptoms
Dystonia
Body distribution of dystonia
Craniofacial dystonia
Upper face dystonia
Lower face dystonia
Oromandibular dystonia
Jaw dystonia
Laryngeal dystonia
Limb dystonia
Arm dystonia
Hand dystonia
Leg dystonia
Foot dystonia

Cervical dystonia
Torticollis
Retrocollis
Axial dystonia
Rest dystonia
Action-induced dystonia
Dystonic Posture
Mirror dystonia
Alleviation maneuvers
Overflow dystonia
Tremor (any or unspecified)
Dystonic tremor
Action tremor
Postural tremor
Resting tremor
Intention tremor
Kinetic tremor
Upper limb postural tremor
Head tremor
Limb tremor
Hand tremor
Alcohol responsiveness
Motor instrument (e.g. GDRS, FMRS motor and disability score, or TWSTRS) and, if available, their scoring
Dystonia onset body part
Dystonia onset side
Distribution of dystonia (focal, segmental, multifocal, hemidystonia, generalized)
Type of dystonia (isolated, combined or complex)
Pattern of disease course (static or progressive)
Pattern in general (persistent, action-specific, diurnal fluctuations, paroxysmal)
Diurnal fluctuations as motor symptom fluctuations throughout the day
Motor fluctuations in context of medication intake
Sleep benefit as restoration of mobility upon awakening from sleep
Parkinsonism
Bradykinesia
Rigidity
Postural instability
Muscular hypotonia
Spasticity
Hyperreflexia

Dyskinesia
Seizures
Dysarthria
Anarthria
Dysphonia
Response to treatment, quantified as none, positive, negative or temporary
Botulinum toxin
Anticholinergics
Baclofen (intrathecal)
Benzodiazepines
VMAT2 inhibitors
Dopaminergic drugs
Amantadine
Other medication
Stereotactic surgery (e.g. DBS, thalamotomy or pallidotomy)
Non-motor signs and symptoms
Developmental regression
Dysmorphic features
Microcephaly
Short stature
Decreased body weight
Intellectual deficit
Global developmental delay
Motor delay
Cognitive decline
Delay of speech and language development
Depression
Anxiety
Psychotic symptoms
Sleep disorder
Autonomic symptoms
Abnormality of the sense of smell
**Genetic information**
Genetic status of all tested family members with number of homozygous, heterozygous and wildtype individuals in affected and not affected state
Gene to which the mutation refers
Genome build/transcript (ESTN number, NCBI number)
Physical location of the mutation (on the plus strand)
Reference bases on the plus strand
Observed mutated bases on the plus strand

Genomic description of the mutation according to the nomenclature of the HGVS
Coding description of the mutation according to the nomenclature of the HGVS
Protein description of the mutation according to the nomenclature of the HGVS
Genotype of the respective mutation
Type of mutation (missense. nonsense. in-frame indel. frameshift. splice site. structural variation)
Sporadic mutation/de-novo status
Highest MAF of the respective mutation found in gnomad
Pathogenicity status classified as benign. possibly. probably or definitely pathogenic
CADD score
**Gene-specific variables**
GLB1
Upper motor neuron dysfunction
Amyotrophy
Hepatosplenomegaly
Ophthalmologic abnormalities
Abnormality of skeletal morphology
Ataxia
Facial grimacing
Myoclonus
Chorea
Gait disturbance
MRI abnormalities
SLC6A3
Upper motor neuron dysfunction
Orolingual dyskinesia
Saccade initiation failure
Ocular flutter
Oculogyric crisis
Slow saccades
Dystonic crisis
CSF HVA nmol/L
CSF HVA:Hydroxyindoleacetic acid ratio
Chorea
Ptosis
Eyelid fluttering/ myoclonus
SLC30A10 and *SCL39A14*
Hepatomegaly
Splenomegaly
Hepatic cirrhosis
Gait difficulties

Spastic paraparesis
Hypomimia
Wide based gait
Hypermanganesemia
Manganese level
Polycythemia
Hemoglobin level
Iron level
Ferritin level
T1 hyperintensities on MRI
Sensory axonal polyneuropathy
Hyperesthesia
Extensor plantar response
Hyporeflexia
Bowel incontinence
PLA2G6
MRI hypointensity on basal ganglia or substantia nigra
Other MRI abnormalities
DaTSCAN/TRODAT with evidence of dopaminergic deficit

AAO: age at disease onset; GDRS: Global Dystonia Rating Scale; FMRS: Fahn-Marsden Rating Scale; TWSTRS: Toronto Western Spasmodic Torticollis Rating Scale; VMAT2: vesicular monoamine transporter 2; DBS: deep brain stimulation; ESTN: Expressed Sequence Tag Number; NCBI: National Center for Biotechnology Information; HGVS: human genome variation society; MAF: minor allele frequency; CADD: Combined Annotation Dependent Depletion; MRI: magnetic resonance imaging; HVA: homovanillic acid

Table S3. Summary of missing data for each gene

Missing data	n	%
GLB1 (n=67)		
Sex	19	28.4%
Consanguinity	19	28.4%
Age at onset	26	38.8%
Age at diagnosis	24	35.8%
Age at examination	23	34.3%
Initial symptom	20	29.9%
Dystonia	15	22.4%
Body distribution of dystonia	53	79.1%
Craniofacial dystonia	46	68.7%
Ataxia	34	50.7%
Dysarthria/Anarthria	35	52.2%
Dysphagia	25	37.3%
Parkinsonism	50	74.6%
Muscular hypotonia	34	50.7%
Upper motor neuron dysfunction	31	46.3%
Seizures	23	34.3%
Cognitive impairment	45	67.2%
Developmental disorders (delay or regression)	25	37.3%
Hepatosplenomegaly	17	25.4%
Ophthalmologic abnormalities	24	35.8%
Abnormality of skeletal morphology	12	17.9%
Dysmorphic features	39	58.2%
Microcephaly	47	70.1%
Short stature	20	29.9%
SLC6A3 **(**n=25)		
Sex	0	0%
Consanguinity	3	12%
Age at onset	3	12%
Age at diagnosis	9	36%
Age at examination	1	4%
Initial symptom	6	24%
Dystonia	1	4%
Body distribution of dystonia	2	8%
Oromandibular dystonia	14	56%
Status dystonicus	15	60%
Dysarthria/Anarthria	13	52%
Parkinsonism	4	16%

Resting tremor	12	48%
Muscular hypotonia	11	44%
Irritability and feeding problems	17	68%
Dyskinesia	13	52%
Orolingual dyskinesia	16	64%
Upper motor neuron dysfunction	15	60%
Saccade initiation failure	18	72%
Ocular flutter	16	64%
Oculogyric crisis	19	76%
Slow saccades	18	72%
Cognitive impairment	21	84%
Developmental disorders (delay or regression)	1	4%
CSF HVA:Hydroxyindoleacetic acid ratio	9	36%
Response to dopaminergic treatment	6	24%
***SLC30A10* (n=52)**		
Sex	2	3.8%
Ethnicity	18	34.6%
Consanguinity	0	0%
Age at onset	0	0%
Age at diagnosis	44	84.6%
Age at examination	0	0%
Initial symptom	13	25%
Dystonia	1	1.9%
Body distribution of dystonia	17	32.7%
Dystonia onset body part	33	63.5%
Dysarthria/Anarthria	27	51.9%
Parkinsonism	31	59.6%
Gait difficulties	23	44.2%
Cognitive impairment	20	38.5%
Developmental disorders (delay or regression)	27	51.9%
Hypermanganesemia	1	1.9%
Polycythemia	1	1.9%
Ferritin levels	23	44.2%
T1 hyperintensities, basal ganglia	4	7.7%
Response to dissodium calcium edetate	34	65.4%
Response to ethylenediaminetetraacetic acid	47	90.4%
Response to penicillamine	47	90.4%
Response to dopaminergic treatment	36	69.2%
***SLC39A14* (n=22)**		
Sex	1	4.5%

Country of origin	1	4.5%
Consanguinity	2	9.1%
Age at onset	4	18.2%
Age at diagnosis	12	54.5%
Age at examination	1	4.5%
Initial symptom	2	9.1%
Dystonia	0	0%
Body distribution of dystonia	8	36.4%
Dystonia onset body part	16	72.7%
Dysarthria/Anarthria	10	45.5%
Parkinsonism	17	77.3%
Hyperreflexia	11	50%
Spasticity	11	50%
Tip-toe gait	16	72.7%
Depression, anxiety or psychosis	21	95.5%
Cognitive impairment	12	54.5%
Developmental disorders (delay or regression)	7	31.8%
Microcephaly	14	63.6%
Hepatomegaly	43	82.7%
Cirrhosis	30	57.7%
Hypermanganesemia	2	9.1%
Polycythemia	7	31.8%
Ferritin levels	17	77.3%
T1 hyperintensities, basal ganglia	2	9.1%
T1 hyperintensities, pituitary gland	15	68.2%
Response to dissodium calcium edetate	12	54.5%
Response to intrathecal baclofen	20	90.1%
Response to anticholinergics	15	68.2%
Response to dopaminergic treatment	15	68.2%
Response to benzodiazepines	15	68.2%
***PLA2G6* (n=220)**		
Sex	10	4.5%
Country of origin	47	21.4%
Consanguinity	42	19.1%
Age at onset	32	14.5%
Age at diagnosis	139	63.2%
Age at examination	34	15.5%
Initial symptom	105	47.7%
Dystonia	74	33.6%
Body distribution of dystonia	158	71.8%

Craniofacial dystonia	156	70.9%
Ataxia	116	52.7%
Dysarthria/Anarthria	147	66.8%
Parkinsonism	83	37.7%
Resting tremor	151	68.6%
Motor fluctuations	199	90.5%
Dyskinesia	139	63.2%
Muscular hypotonia	129	58.6%
Upper motor neuron dysfunction	75	34.1%
Seizures	105	47.7%
Cognitive impairment	61	27.7%
Developmental disorders (delay or regression)	97	44.1%
Depression, anxiety or psychosis	133	60.5%
Ocular abnormalities	101	45.9%
MRI, abnormal	35	15.9%
Cerebellar atrophy	43	19.5%
MRI hypointensities, basal ganglia or substantia nigra	48	21.8%
DaTSCAN/TRODAT with evidence of dopaminergic deficit	172	78.2%
Response to amantadine	207	94.1%
Response to oral baclofen	216	98.2%
Response to dopaminergic treatment	130	59.1%
Response to deep-brain stimulation	213	96.8%

Footnotes. HVA: homovanillic acid; MRI: magnetic resonance imaging

Table S4. List of all the included articles

Included articles
GLB1
1. Arash-Kaps L, Komlosi K, Seegräber M, Diederich S, Paschke E, Amraoui Y, et al. The Clinical and Molecular Spectrum of GM1 Gangliosidosis. J Pediatr. 2019 Dec;215:152-157.e3. doi: 10.1016/j.jpeds.2019.08.016.
2. Caciotti A, Donati MA, Bardelli T, d'Azzo A, Massai G, Luciani L, et al. Primary and secondary elastin-binding protein defect leads to impaired elastogenesis in fibroblasts from GM1-gangliosidosis patients. Am J Pathol. 2005 Dec;167(6):1689-98. doi: 10.1016/S0002-9440(10)61251-5.
3. Caciotti A, Garman SC, Rivera-Colón Y, Procopio E, Catarzi S, Ferri L, et al. GM1 gangliosidosis and Morquio B disease: an update on genetic alterations and clinical findings. Biochim Biophys Acta. 2011 Jul;1812(7):782-90. doi: 10.1016/j.bbadis.2011.03.018.
4. Chakraborty S, Rafi MA, Wenger DA. Mutations in the lysosomal beta-galactosidase gene that cause the adult form of GM1 gangliosidosis. Am J Hum Genet. 1994 Jun;54(6):1004-13.
5. Deodato F, Procopio E, Rampazzo A, Taurisano R, Donati MA, Dionisi-Vici C, et al. The treatment of juvenile/adult GM1-gangliosidosis with Miglustat may reverse disease progression. Metab Brain Dis. 2017 Oct;32(5):1529-1536. doi: 10.1007/s11011-017-0044-y.
6. Emecen Sanli M, Dogan M. GM1 gangliosidosis: patients with different phenotypic features and novel mutations. J Pediatr Endocrinol Metab. 2023 Apr 13;36(6):602-607. doi: 10.1515/jpem-2022-0630.
7. Giugliani L, Steiner CE, Kim CA, Lourenço CM, Santos MLSF, de Souza CFM, et al. Clinical findings in Brazilian patients with adult GM1 gangliosidosis. JIMD Rep. 2019 Jul 17;49(1):96-106. doi: 10.1002/jmd2.12067.
8. Hajirnis O, Udwadia-Hegde A. Chronic GM1 Gangliosidosis with Characteristic "Wish Bone Sign" on Brain MRI. Another Type of Neurodegeneration with Brain Iron Accumulation? Mov Disord Clin Pract. 2015 Jun 2;2(3):323-325. doi: 10.1002/mdc3.12197.
9. Hirayama M, Kitagawa Y, Yamamoto S, Tokuda A, Mutoh T, Hamano T, et al. GM1 gangliosidosis type 3 with severe jaw-closing impairment. J Neurol Sci. 1997 Nov 6;152(1):99-101. doi: 10.1016/s0022-510x(97)00139-1.
10. Kaiyrzhanov R, Guliyeva U, Gulieva S, Salayev K, Mursalova A, Allahyarova P, et al. GM1-Gangliosidosis Type III Associated Parkinsonism. Mov Disord Clin Pract. 2021 Sep 3;8(Suppl 1):S21-S23. doi: 10.1002/mdc3.13289.
11. Kannebley JS, Silveira-Moriyama L, Bastos LO, Steiner CE. Clinical Findings and Natural History in Ten Unrelated Families with Juvenile and Adult GM1 Gangliosidosis. JIMD Rep. 2015;24:115-22. doi: 10.1007/8904_2015_451.
12. Koya Kutty S, Magrinelli F, Milner AV, Bhatia KP. Abnormal DaTscan in GM1-Gangliosidosis Type III Manifesting with Dystonia-Parkinsonism. Mov Disord Clin Pract. 2022 Jul 10;9(6):825-828. doi: 10.1002/mdc3.13512.

13. Kumar KR, Davis RL, Tchan MC, Wali GM, Mahant N, Ng K, et al. Whole genome sequencing for the genetic diagnosis of heterogenous dystonia phenotypes. Parkinsonism Relat Disord. 2019 Dec;69:111-118. doi: 10.1016/j.parkreldis.2019.11.004.
14. Martínez-Rubio D, Hinarejos I, Sancho P, Gorría-Redondo N, Bernadó-Fonz R, Tello C, et al. Mutations, Genes, and Phenotypes Related to Movement Disorders and Ataxias. Int J Mol Sci. 2022 Oct 6;23(19):11847. doi: 10.3390/ijms231911847.
15. Pierson TM, Adams DA, Markello T, Golas G, Yang S, Sincan M, et al. Exome sequencing as a diagnostic tool in a case of undiagnosed juvenile-onset GM1-gangliosidosis. Neurology. 2012 Jul 10;79(2):123-6. doi: 10.1212/WNL.0b013e31825f047a.
16. Roze E, Paschke E, Lopez N, Eck T, Yoshida K, Maurel-Ollivier A, et al. Dystonia and parkinsonism in GM1 type 3 gangliosidosis. Mov Disord. 2005 Oct;20(10):1366-9. doi: 10.1002/mds.20593.
17. Stockler-Ipsiroglu S, Yazdanpanah N, Yazdanpanah M, Moisa Popurs M, Yuskiv N, Schmitz Ferreira Santos ML, et al. Morquio-like dysostosis multiplex presenting with neuronopathic features is a distinct *GLB1*-related phenotype. JIMD Rep. 2021 Mar 8;60(1):23-31. doi: 10.1002/jmd2.12211.
18. Takenouchi T, Kosaki R, Nakabayashi K, Hata K, Takahashi T, Kosaki K. Paramagnetic signals in the globus pallidus as late radiographic sign of juvenile-onset GM1 gangliosidosis. Pediatr Neurol. 2015 Feb;52(2):226-9. doi: 10.1016/j.pediatrneurol.2014.09.022.
19. Tanaka R, Momoi T, Yoshida A, Okumura M, Yamakura S, Takasaki Y, et al. Type 3 GM1 gangliosidosis: clinical and neuroradiological findings in an 11-year-old girl. J Neurol. 1995 May;242(5):299-303. doi: 10.1007/BF00878872.
20. Tebani A, Sudrié-Arnaud B, Dabaj I, Torre S, Domitille L, Snanoudj S, et al. Disentangling molecular and clinical stratification patterns in beta-galactosidase deficiency. J Med Genet. 2022 Apr;59(4):377-384. doi: 10.1136/jmedgenet-2020-107510.
21. Vieira JP, Conceição C, Scortenschi E. GM1 gangliosidosis, late infantile onset dystonia, and T2 Hypointensity in the globus pallidus and substantia Nigra. Pediatr Neurol. 2013 Sep;49(3):195-7. doi: 10.1016/j.pediatrneurol.2013.02.003.

SLC6A3

1. Baga M, Spagnoli C, Soliani L, Salerno GG, Rizzi S, Frattini D, et al. Early-onset Dopamine Transporter Deficiency Syndrome: Long-term Follow-up. Can J Neurol Sci. 2021 Mar;48(2):285-286. doi: 10.1017/cjn.2020.144.
2. Hansen FH, Skjørringe T, Yasmeen S, Arends NV, Sahai MA, Erreger K, et al. Missense dopamine transporter mutations associate with adult parkinsonism and ADHD. J Clin Invest. 2014 Jul;124(7):3107-20. doi: 10.1172/JCI73778.
3. Heidari E, Razmara E, Hosseinpour S, Tavasoli AR, Garshasbi M. Homozygous in-frame variant of SCL6A3 causes dopamine transporter deficiency syndrome in a consanguineous family. Ann Hum Genet. 2020 Jul;84(4):315-323. doi: 10.1111/ahg.12378.
4. Kurian MA, Zhen J, Cheng SY, Li Y, Mordekar SR, Jardine P, et al. Homozygous loss-of-function mutations in the gene encoding the dopamine transporter are associated with infantile parkinsonism-dystonia. J Clin Invest. 2009 Jun;119(6):1595-603. doi: 10.1172/JCI39060.

5. Kurian MA, Li Y, Zhen J, Meyer E, Hai N, Christen HJ, et al. Clinical and molecular characterisation of hereditary dopamine transporter deficiency syndrome: an observational cohort and experimental study. Lancet Neurol. 2011 Jan;10(1):54-62. doi: 10.1016/S1474-4422(10)70269-6.
6. Mir A, Almudhry M, Alghamdi F, Albaradie R, Ibrahim M, Aldurayhim F, et al. SLC gene mutations and pediatric neurological disorders: diverse clinical phenotypes in a Saudi Arabian population. Hum Genet. 2022 Jan;141(1):81-99. doi: 10.1007/s00439-021-02404-x.
7. Nasehi MM, Nikkhah A, Salari M, Soltani P, Shirzadi S. Dopamine Transporter Deficiency Syndrome: A Case with Hyper- and Hypokinetic Extremes. Mov Disord Clin Pract. 2020 Sep 29;7(Suppl 3):S57-S60. doi: 10.1002/mdc3.13064.
8. Ng J, Zhen J, Meyer E, Erreger K, Li Y, Kakar N, et al. Dopamine transporter deficiency syndrome: phenotypic spectrum from infancy to adulthood. Brain. 2014 Apr;137(Pt 4):1107-19. doi: 10.1093/brain/awu022.
9. Puffenberger EG, Jinks RN, Sougnez C, Cibulskis K, Willert RA, Achilly NP, et al. Genetic mapping and exome sequencing identify variants associated with five novel diseases. PLoS One. 2012;7(1):e28936. doi: 10.1371/journal.pone.0028936.
10. Yildiz Y, Pektas E, Tokatli A, Haliloglu G. Hereditary Dopamine Transporter Deficiency Syndrome: Challenges in Diagnosis and Treatment. Neuropediatrics. 2017 Feb;48(1):49-52. doi: 10.1055/s-0036-1593372.
11. Zemojtel T, Köhler S, Mackenroth L, Jäger M, Hecht J, Krawitz P, et al. Effective diagnosis of genetic disease by computational phenotype analysis of the disease-associated genome. Sci Transl Med. 2014 Sep 3;6(252):252ra123. doi: 10.1126/scitranslmed.3009262.

SLC30A10

1. Cherian A, Priya L, Divya KP. "Cock-walk" gait and "horseshoe moustache" sign on MRI in inherited hypermanganesemia. Neurol Sci. 2022 Feb;43(2):1441-1445. doi: 10.1007/s10072-021-05793-z.
2. Dutta A, Majumdar R, Dubey S, Pandit A. Penicillamine for Hypermanganesemia With Dystonia, Polycythemia, and Cirrhosis in 2 Sisters. Neurology. 2021 Jan 19;96(3):123-125. doi: 10.1212/WNL.0000000000011296.
3. Garg D, Yoganathan S, Shamim U, Mankad K, Gulati P, Bonifati V, et al. Clinical Profile and Treatment Outcomes of Hypermanganesemia with Dystonia 1 and 2 among 27 Indian Children. Mov Disord Clin Pract. 2022 Aug 12;9(7):886-899. doi: 10.1002/mdc3.13516.
4. Gulab S, Kayyali HR, Al-Said Y. Atypical Neurologic Phenotype and Novel SLC30A10 Mutation in Two Brothers with Hereditary Hypermanganesemia. Neuropediatrics. 2018 Feb;49(1):72-75. doi: 10.1055/s-0037-1608778.
5. Lambrianides S, Nicolaou P, Michaelidou M, Kakouris P, Votsi C, Petrou PP, et al. A novel SLC30A10 missense variant associated with parkinsonism and dystonia without hypermanganesemia. J Neurol Sci. 2020 Nov 15;418:117101. doi: 10.1016/j.jns.2020.117101.
6. Mukhtiar K, Ibrahim S, Tuschl K, Mills P. Hypermanganesemia with Dystonia, Polycythemia and Cirrhosis (HMDPC) due to mutation in the SLC30A10 gene. Brain Dev. 2016 Oct;38(9):862-5. doi: 10.1016/j.braindev.2016.04.005.

7. Padmanabha H, Krishnamurthy S, Sharath Kumar GG, Chikkanayakana I, Sethuraman A, Mathew T. Teaching NeuroImages: An imaging clue for treatable early childhood-onset dystonia: Manganism. Neurology. 2019 Feb 5;92(6):e628-e629. doi: 10.1212/WNL.0000000000006881.
8. Quadri M, Federico A, Zhao T, Breedveld GJ, Battisti C, Delnooz C, et al. Mutations in SLC30A10 cause parkinsonism and dystonia with hypermanganesemia, polycythemia, and chronic liver disease. Am J Hum Genet. 2012 Mar 9;90(3):467-77. doi: 10.1016/j.ajhg.2012.01.017.
9. Quadri M, Kamate M, Sharma S, Olgiati S, Graafland J, Breedveld GJ, et al. Manganese transport disorder: novel SLC30A10 mutations and early phenotypes. Mov Disord. 2015 Jun;30(7):996-1001. doi: 10.1002/mds.26202.
10. Santhakumar S, Lukas J, Unnikrishnan G, Thomas B, Kesavadas C. Treatable Hereditary Manganese Transport Disorder: Novel *SLC30A10* Mutation and its Characteristic Neuroimaging Appearance in Two Siblings. J Pediatr Genet. 2020 Jul 23;10(4):305-310. doi: 10.1055/s-0040-1713853.
11. Tabatabaee SN, Effat Nejad S, Nikkhah A, Hashemi N, Alavi A, Lang AE, et al. Familial Hypermanganesemia in Iran. Mov Disord Clin Pract. 2023 Mar 26;10(5):850-853. doi: 10.1002/mdc3.13723.
12. Tavasoli A, Arjmandi Rafsanjani K, Hemmati S, Mojbafan M, Zarei E, Hosseini S. A case of dystonia with polycythemia and hypermanganesemia caused by SLC30A10 mutation: a treatable inborn error of manganese metabolism. BMC Pediatr. 2019 Jul 9;19(1):229. doi: 10.1186/s12887-019-1611-7.
13. Tuschl K, Clayton PT, Gospe SM Jr, Gulab S, Ibrahim S, Singhi P, et al. Syndrome of hepatic cirrhosis, dystonia, polycythemia, and hypermanganesemia caused by mutations in SLC30A10, a manganese transporter in man. Am J Hum Genet. 2012 Mar 9;90(3):457-66. doi: 10.1016/j.ajhg.2012.01.018.
14. Yapici Z, Tuschl K, Eraksoy M. Hypermanganesemia with Dystonia 1: A Novel Mutation and Response to Iron Supplementation. Mov Disord Clin Pract. 2019 Nov 12;7(1):94-96. doi: 10.1002/mdc3.12861.
15. Zaki MS, Issa MY, Elbendary HM, El-Karaksy H, Hosny H, Ghobrial C, et al. Hypermanganesemia with dystonia, polycythemia and cirrhosis in 10 patients: Six novel SLC30A10 mutations and further phenotype delineation. Clin Genet. 2018 Apr;93(4):905-912. doi: 10.1111/cge.13184.

SLC39A14

1. Alhasan KA, Alshuaibi W, Hamad MH, Salim S, Jamjoom DZ, Alhashim AH, et al. Hypermanganesemia with Dystonia Type 2: A Potentially Treatable Neurodegenerative Disorder: A Case Series in a Tertiary University Hospital. Children (Basel). 2022 Sep 1;9(9):1335. doi: 10.3390/children9091335.
2. Juneja M, Shamim U, Joshi A, Mathur A, Uppili B, Sairam S, et al. A novel mutation in SLC39A14 causing hypermanganesemia associated with infantile onset dystonia. J Gene Med. 2018 Apr;20(4):e3012. doi: 10.1002/jgm.3012.
3. Lee JH, Shin JH. Effect of Chelation Therapy on a Korean Patient With Brain Manganese Deposition Resulting From a Compound Heterozygous Mutation in the SLC39A14 Gene. J Mov Disord. 2022 May;15(2):171-174. doi: 10.14802/jmd.21143.

4. Marti-Sanchez L, Ortigoza-Escobar JD, Darling A, Villaronga M, Baide H, Molero-Luis M, et al. Hypermanganesemia due to mutations in SLC39A14: further insights into Mn deposition in the central nervous system. Orphanet J Rare Dis. 2018 Jan 30;13(1):28. doi: 10.1186/s13023-018-0758-x.
5. Namnah M, Bauer M, Mor-Shaked H, Bressman SB, Raymond D, Ozelius LJ, et al. Benign SLC39A14 Course of Dystonia-Parkinsonism Secondary to Inherited Manganese Accumulation. Mov Disord Clin Pract. 2020 May 7;7(5):569-570. doi: 10.1002/mdc3.12947.
6. Rodan LH, Hauptman M, D'Gama AM, Qualls AE, Cao S, Tuschl K, et al. Novel founder intronic variant in SLC39A14 in two families causing Manganism and potential treatment strategies. Mol Genet Metab. 2018 Jun;124(2):161-167. doi: 10.1016/j.ymgme.2018.04.002.
7. Tabatabaee SN, Effat Nejad S, Nikkhah A, Hashemi N, Alavi A, Lang AE, et al. Familial Hypermanganesemia in Iran. Mov Disord Clin Pract. 2023 Mar 26;10(5):850-853. doi: 10.1002/mdc3.13723.
8. Tuschl K, Meyer E, Valdivia LE, Zhao N, Dadswell C, Abdul-Sada A, et al. Mutations in SLC39A14 disrupt manganese homeostasis and cause childhood-onset parkinsonism-dystonia. Nat Commun. 2016 May 27;7:11601. doi: 10.1038/ncomms11601.
9. Zeglam A, Abugrara A, Kabuka M. Autosomal-recessive iron deficiency anemia, dystonia and hypermanganesemia caused by new variant mutation of the manganese transporter gene SLC39A14. Acta Neurol Belg. 2019 Sep;119(3):379-384. doi: 10.1007/s13760-018-1024-7.
10. Zhang M, Zhu L, Wang H, Hao Y, Zhang Q, Zhao C, et al. A novel homozygous *SLC39A14* variant in an infant with hypermanganesemia and a review of the literature. Front Pediatr. 2023 Jan 17;10:949651. doi: 10.3389/fped.2022.949651.

PLA2G6

1. Agarwal P, Hogarth P, Hayflick S, MacLeod P, Kuriakose R, McKenzie J, et al. Imaging striatal dopaminergic function in phospholipase A2 group VI-related parkinsonism. Mov Disord. 2012 Nov;27(13):1698-9. doi: 10.1002/mds.25160.
2. Ahn H, Moon HJ, Jeon B. A Case of *PLA2G6*-Associated Neurodegeneration with Frequent Myoclonus And Generalized Onset Tonic-Clonic Seizures: Successful Treatment with Zonisamide. J Clin Neurol. 2021 Apr;17(2):319-321. doi: 10.3988/jcn.2021.17.2.319.
3. Al-Maawali A, Yoon G, Feigenbaum AS, Halliday WC, Clarke JT, Branson HM, et al. Validation of the finding of hypertrophy of the clava in infantile neuroaxonal dystrophy/PLA2G6 by biometric analysis. Neuroradiology. 2016 Oct;58(10):1035-1042. doi: 10.1007/s00234-016-1726-6.
4. Bakhit Y, Tesson C, Ibrahim MO, Eltom K, Eltazi I, Elsayed LEO, et al. PLA2G6-associated late-onset parkinsonism in a Sudanese family. Ann Clin Transl Neurol. 2023 Jun;10(6):983-989. doi: 10.1002/acn3.51781.
5. Bhardwaj NK, Gowda VK, Saini J, Sardesai AV, Santhoshkumar R, Mahadevan A. Neurodegeneration with brain iron accumulation: Characterization of clinical, radiological, and genetic features of pediatric patients from Southern India. Brain Dev. 2021 Nov;43(10):1013-1022. doi: 10.1016/j.braindev.2021.06.010.

6. Blake RB, Gilbert DL, Schapiro MB. Child Neurology: Two sisters with dystonia and regression: PLA2G6-associated neurodegeneration. Neurology. 2016 Jul 5;87(1):e1-3. doi: 10.1212/WNL.0000000000002804.
7. Bohlega SA, Al-Mubarak BR, Alyemni EA, Abouelhoda M, Monies D, Mustafa AE, et al. Clinical heterogeneity of PLA2G6-related Parkinsonism: analysis of two Saudi families. BMC Res Notes. 2016 Jun 7;9:295. doi: 10.1186/s13104-016-2102-7.
8. Chen YJ, Chen YC, Dong HL, Li LX, Ni W, Li HF, et al. Novel PLA2G6 mutations and clinical heterogeneity in Chinese cases with phospholipase A2-associated neurodegeneration. Parkinsonism Relat Disord. 2018 Apr;49:88-94. doi: 10.1016/j.parkreldis.2018.02.010.
9. Chen S, Zhang H, Zhang J, Jiang B, He Z, Zhang B, et al. Motor and non-motor responses of STN DBS in early onset PLA2G6 related Parkinsonism with compound heterozygous mutation from China. Parkinsonism Relat Disord. 2023 Jan;106:105237. doi: 10.1016/j.parkreldis.2022.105237.
10. Cheng HL, Chen YJ, Xue YY, Wu ZY, Li HF, Wang N. Clinical Characterization and Founder Effect Analysis in Chinese Patients with Phospholipase A2-Associated Neurodegeneration. Brain Sci. 2022 Apr 19;12(5):517. doi: 10.3390/brainsci12050517.
11. Chu YT, Lin HY, Chen PL, Lin CH. Genotype-phenotype correlations of adult-onset PLA2G6-associated Neurodegeneration: case series and literature review. BMC Neurol. 2020 Mar 17;20(1):101. doi: 10.1186/s12883-020-01684-6.
12. Cif L, Kurian MA, Gonzalez V, Garcia-Ptacek S, Roujeau T, Gelisse P, et al. Atypical *PLA2G6*-Associated Neurodegeneration: Social Communication Impairment, Dystonia and Response to Deep Brain Stimulation. Mov Disord Clin Pract. 2014 May 23;1(2):128-131. doi: 10.1002/mdc3.12030.
13. Crompton D, Rehal PK, MacPherson L, Foster K, Lunt P, Hughes I, et al. Multiplex ligation-dependent probe amplification (MLPA) analysis is an effective tool for the detection of novel intragenic PLA2G6 mutations: implications for molecular diagnosis. Mol Genet Metab. 2010 Jun;100(2):207-12. doi: 10.1016/j.ymgme.2010.02.009.
14. Darling A, Aguilera-Albesa S, Tello CA, Serrano M, Tomás M, Camino-León R, et al. PLA2G6-associated neurodegeneration: New insights into brain abnormalities and disease progression. Parkinsonism Relat Disord. 2019 Apr;61:179-186. doi: 10.1016/j.parkreldis.2018.10.013.
15. de Oliveira P, Montanaro V, Carvalho D, Martins B, Ferreira A, Cardoso F. Severe Early-Onset Parkinsonian Syndrome Caused by *PLA2G6* Heterozygous Variants. Mov Disord Clin Pract. 2021 May 6;8(5):794-796. doi: 10.1002/mdc3.13230.
16. Dehnavi AZ, Bemanalizadeh M, Kahani SM, Ashrafi MR, Rohani M, Toosi MB, et al. Phenotype and genotype heterogeneity of PLA2G6-associated neurodegeneration in a cohort of pediatric and adult patients. Orphanet J Rare Dis. 2023 Jul 5;18(1):177. doi: 10.1186/s13023-023-02780-9.
17. Demir Unal E, Kaplan H, Dirik EB. Psychoradiological evaluation of PLA2G6 gene mutations: Adult-onset dystonia-parkinsonism case report. Parkinsonism Relat Disord. 2022 Oct;103:150-151. doi: 10.1016/j.parkreldis.2022.09.004.
18. Erro R, Balint B, Kurian MA, Brugger F, Picillo M, Barone P, et al. Early Ataxia and Subsequent Parkinsonism: PLA2G6 Mutations Cause a Continuum Rather Than Three Discrete Phenotypes. Mov Disord Clin Pract. 2016 Mar 31;4(1):125-128. doi: 10.1002/mdc3.12319.

19. Gafner M, Michelson M, Yosovich K, Blumkin L, Lerman-Sagie T, Lev D. Infantile onset progressive cerebellar atrophy and anterior horn cell Degeneration-A novel phenotype associated with mutations in the PLA2G6 gene. Eur J Med Genet. 2020 Apr;63(4):103801. doi: 10.1016/j.ejmg.2019.103801.
20. Gao L, Shi C, Lin Q, Wu Y, Hu L, Wang M, et al. Case Report: A case of *PLA2G6*gene-related early-onset Parkinson's disease and review of literature. Front Neurosci. 2022 Dec 9;16:1064566. doi: 10.3389/fnins.2022.1064566.
21. Guo S, Yang L, Liu H, Chen W, Li J, Yu P, et al. Identification of Novel Compound Mutations in PLA2G6-Associated Neurodegeneration Patient with Characteristic MRI Imaging. Mol Neurobiol. 2017 Aug;54(6):4636-4643. doi: 10.1007/s12035-016-9991-2.
22. Hanna Al-Shaikh R, Milanowski LM, Holla VV, Kurihara K, Yadav R, Kamble N, et al. PLA2G6-associated neurodegeneration in four different populations-case series and literature review. Parkinsonism Relat Disord. 2022 Aug;101:66-74. doi: 10.1016/j.parkreldis.2022.06.016.
23. Hao X, Yang Q, Shen L, Du J. A case of neurodegenerative disorder caused by PLA2G6 compound heterozygous pathogenic variant and SCA17 pathogenic variant. Neurol Sci. 2022 Mar;43(3):2105-2108. doi: 10.1007/s10072-021-05821-y.
24. Hua P, Zhao Y, Zeng Q, Li L, Ren J, Guo J, et al. Genetic Analysis of Patients With Early-Onset Parkinson's Disease in Eastern China. Front Aging Neurosci. 2022 May 11;14:849462. doi: 10.3389/fnagi.2022.849462.
25. Huang MH, Chiu YC, Tsai CF. Aripiprazole in a Patient of PLA2G6-Associated Neurodegeneration With Psychosis. Clin Neuropharmacol. 2018 Jul/Aug;41(4):136-137. doi: 10.1097/WNF.0000000000000284.
26. Illingworth MA, Meyer E, Chong WK, Manzur AY, Carr LJ, Younis R, et al. PLA2G6-associated neurodegeneration (PLAN): further expansion of the clinical, radiological and mutation spectrum associated with infantile and atypical childhood-onset disease. Mol Genet Metab. 2014 Jun;112(2):183-9. doi: 10.1016/j.ymgme.2014.03.008.
27. Jain S, Bhasin H, Romani M, Valente EM, Sharma S. Atypical Childhood-onset Neuroaxonal Dystrophy in an Indian Girl. J Pediatr Neurosci. 2019 Apr-Jun;14(2):90-93. doi: 10.4103/jpn.JPN_91_18.
28. Ji Y, Li Y, Shi C, Gao Y, Yang J, Liang D, et al. Identification of a novel mutation in PLA2G6 gene and phenotypic heterogeneity analysis of PLA2G6-related neurodegeneration. Parkinsonism Relat Disord. 2019 Aug;65:159-164. doi: 10.1016/j.parkreldis.2019.04.002.
29. Jiao B, Zhou Z, Hu Z, Du J, Liao X, Luo Y, et al. Homozygosity mapping and next generation sequencing for the genetic diagnosis of hereditary ataxia and spastic paraplegia in consanguineous families. Parkinsonism Relat Disord. 2020 Nov;80:65-72. doi: 10.1016/j.parkreldis.2020.09.013.
30. Kamel WA, Al-Hashel JY, Abdulsalam AJ, Damier P, Al-Mejalhem AY. PLA2G6-related parkinsonism presenting as adolescent behavior. Acta Neurol Belg. 2019 Dec;119(4):621-622. doi: 10.1007/s13760-018-1003-z.
31. Kapoor S, Shah MH, Singh N, Rather MI, Bhat V, Gopinath S, et al. Genetic Analysis of PLA2G6 in 22 Indian Families with Infantile Neuroaxonal Dystrophy, Atypical Late-Onset Neuroaxonal

Dystrophy and Dystonia Parkinsonism Complex. PLoS One. 2016 May 19;11(5):e0155605. doi: 10.1371/journal.pone.0155605.

32. Karkheiran S, Shahidi GA, Walker RH, Paisán-Ruiz C. PLA2G6-associated Dystonia-Parkinsonism: Case Report and Literature Review. Tremor Other Hyperkinet Mov (N Y). 2015 Jul 10;5:317. doi: 10.7916/D84Q7T4W.
33. Kim YJ, Lyoo CH, Hong S, Kim NY, Lee MS. Neuroimaging studies and whole exome sequencing of PLA2G6-associated neurodegeneration in a family with intrafamilial phenotypic heterogeneity. Parkinsonism Relat Disord. 2015 Apr;21(4):402-6. doi: 10.1016/j.parkreldis.2015.01.010.
34. Kim A, Kumar KR, Davis RL, Mallawaarachchi AC, Gayevskiy V, Minoche AE, et al. Increased Diagnostic Yield of Spastic Paraplegia with or Without Cerebellar Ataxia Through Whole-Genome Sequencing. Cerebellum. 2019 Aug;18(4):781-790. doi: 10.1007/s12311-019-01038-0.
35. Klein C, Löchte T, Delamonte SM, Braenne I, Hicks AA, Zschiedrich-Jansen K, et al. PLA2G6 mutations and Parkinsonism: Long-term follow-up of clinical features and neuropathology. Mov Disord. 2016 Dec;31(12):1927-1929. doi: 10.1002/mds.26814.
36. Kulkarni SD, Garg M, Sayed R, Patil VA. Two unusual cases of PLA2G6-associated neurodegeneration from India. Ann Indian Acad Neurol. 2016 Jan-Mar;19(1):115-8. doi: 10.4103/0972-2327.168641.
37. Li L, Fong CY, Tay CG, Tae SK, Suzuki H, Kosaki K, et al. Infantile neuroaxonal dystrophy in a pair of Malaysian siblings with progressive cerebellar atrophy: Description of an expanded phenotype with novel PLA2G6 variants. J Clin Neurosci. 2020 Jan;71:289-292. doi: 10.1016/j.jocn.2019.08.111.
38. Liu Q, Jiang B, Zou M, Wan HJ, Yu ZW, Wang J, et al. A genetic analysis of Chinese patients with early-onset Parkinson' s disease. Neurosci Lett. 2022 Nov 1;790:136880. doi: 10.1016/j.neulet.2022.136880.
39. Lu CS, Lai SC, Wu RM, Weng YH, Huang CL, Chen RS, et al. PLA2G6 mutations in PARK14-linked young-onset parkinsonism and sporadic Parkinson's disease. Am J Med Genet B Neuropsychiatr Genet. 2012 Mar;159B(2):183-91. doi: 10.1002/ajmg.b.32012.
40. Magrinelli F, Mehta S, Di Lazzaro G, Latorre A, Edwards MJ, Balint B, et al. Dissecting the Phenotype and Genotype of PLA2G6-Related Parkinsonism. Mov Disord. 2022 Jan;37(1):148-161. doi: 10.1002/mds.28807.
41. Magrinelli F, Rajapaksha I, Kobylecki C, Latorre A, Mulroy E, Estevez-Fraga C, et al. Reply to: Juvenile PLA2G6-parkinsonism due to Indian 'Asian' p.R741Q mutation, and response to STN DBS. Mov Disord. 2022 Mar;37(3):658-662. doi: 10.1002/mds.28955.
42. Malaguti MC, Melzi V, Di Giacopo R, Monfrini E, Di Biase E, Franco G, et al. A novel homozygous PLA2G6 mutation causes dystonia-parkinsonism. Parkinsonism Relat Disord. 2015 Mar;21(3):337-9. doi: 10.1016/j.parkreldis.2015.01.001.
43. Martínez-Rubio D, Hinarejos I, Sancho P, Gorría-Redondo N, Bernadó-Fonz R, Tello C, et al. Mutations, Genes, and Phenotypes Related to Movement Disorders and Ataxias. Int J Mol Sci. 2022 Oct 6;23(19):11847. doi: 10.3390/ijms231911847.

44. McMillan HJ, Marshall AE, Venkateswaran S, Hartley T, Warman-Chardon J, Ramani AK, et al. Whole genome sequencing reveals biallelic PLA2G6 mutations in siblings with cerebellar atrophy and cap myopathy. Clin Genet. 2021 May;99(5):746-748. doi: 10.1111/cge.13935.
45. Mehta S, Takkar A, Singh D, Aggarwal A, Lal V. Unusual Presentation of *PLA2G6*-Related Neurodegeneration with Retinal Vasculitis. Mov Disord Clin Pract. 2021 Nov 18;9(1):113-117. doi: 10.1002/mdc3.13364.
46. Michelis JP, Hattingen E, Gaertner FC, Minnerop M, Träber F, Biskup S, et al. Expanded phenotype and hippocampal involvement in a novel compound heterozygosity of adult PLA2G6 associated neurodegeneration (PARK14). Parkinsonism Relat Disord. 2017 Apr;37:111-113. doi: 10.1016/j.parkreldis.2017.01.005.
47. Ozes B, Karagoz N, Schüle R, Rebelo A, Sobrido MJ, Harmuth F, et al. PLA2G6 mutations associated with a continuous clinical spectrum from neuroaxonal dystrophy to hereditary spastic paraplegia. Clin Genet. 2017 Nov;92(5):534-539. doi: 10.1111/cge.13008.
48. Paisan-Ruiz C, Bhatia KP, Li A, Hernandez D, Davis M, Wood NW, et al. Characterization of PLA2G6 as a locus for dystonia-parkinsonism. Ann Neurol. 2009 Jan;65(1):19-23. doi: 10.1002/ana.21415.
49. Paisán-Ruiz C, Li A, Schneider SA, Holton JL, Johnson R, Kidd D, et al. Widespread Lewy body and tau accumulation in childhood and adult onset dystonia-parkinsonism cases with PLA2G6 mutations. Neurobiol Aging. 2012 Apr;33(4):814-23. doi: 10.1016/j.neurobiolaging.2010.05.009. Epub 2010 Jul 21.
50. Pérez-Torre P, Escobar Villalba A, Martínez Ulloa P, Kawiorski M, Jiménez-Escrig A, Bazán E, et al. PLA2G6 -Associated Neurodegeneration: Report of a Novel Mutation in Two Siblings with Strikingly Different Clinical Presentation. Mov Disord Clin Pract. 2016 Nov 4;4(1):129-131. doi: 10.1002/mdc3.12419.
51. Ravat P, Shinde S, Shinde SR, Bangar S, Nayak N, Agarwal PA. Juvenile PLA2G6-Parkinsonism Due to Indian 'Asian' p.R741Q Mutation, and Response to STN DBS. Mov Disord. 2022 Mar;37(3):657-658. doi: 10.1002/mds.28950.
52. Roeben B, Zeltner L, Hagberg GE, Scheffler K, Schöls L, Bender B. Susceptibility-Weighted Imaging Reveals Subcortical Iron Deposition in PLA2G6-associated Neurodegeneration: The "Double Cortex Sign". Mov Disord. 2023 May;38(5):904-906. doi: 10.1002/mds.29364.
53. Rohani M, Shahidi G, Vali F, Lang AE, Slow E, Gahl WA, et al. Oculogyric crises in PLA2G6 associated neurodegeneration. Parkinsonism Relat Disord. 2018 Jul;52:111-112. doi: 10.1016/j.parkreldis.2018.03.010.
54. Romani M, Kraoua I, Micalizzi A, Klaa H, Benrhouma H, Drissi C, et al. Infantile and childhood onset PLA2G6-associated neurodegeneration in a large North African cohort. Eur J Neurol. 2015 Jan;22(1):178-86. doi: 10.1111/ene.12552.
55. Sait H, Srivastava S, Pandey M, Ravichandran D, Shukla A, Mandal K, et al. Neurodegeneration with brain iron accumulation: a case series highlighting phenotypic and genotypic diversity in 20 Indian families. Neurogenetics. 2023 Apr;24(2):113-127. doi: 10.1007/s10048-023-00712-0.

56. Sakhardande KA, Reddi VSK, Mishra S, Navin K, Ramu A, Arunachal G, et al. Homozygous PLA2G6 (PARK 14) gene mutation associated neuropsychiatric phenotypes from southern India. Parkinsonism Relat Disord. 2021 Sep;90:49-51. doi: 10.1016/j.parkreldis.2021.07.026.
57. Salih MA, Mundwiller E, Khan AO, AlDrees A, Elmalik SA, Hassan HH, et al. New findings in a global approach to dissect the whole phenotype of PLA2G6 gene mutations. PLoS One. 2013 Oct 9;8(10):e76831. doi: 10.1371/journal.pone.0076831.
58. Shen T, Hu J, Jiang Y, Zhao S, Lin C, Yin X, et al. Early-Onset Parkinson's Disease Caused by PLA2G6 Compound Heterozygous Mutation, a Case Report and Literature Review. Front Neurol. 2019 Aug 21;10:915. doi: 10.3389/fneur.2019.00915.
59. Shi CH, Tang BS, Wang L, Lv ZY, Wang J, Luo LZ, Set al. PLA2G6 gene mutation in autosomal recessive early-onset parkinsonism in a Chinese cohort. Neurology. 2011 Jul 5;77(1):75-81. doi: 10.1212/WNL.0b013e318221acd3.
60. Sina F, Shojaee S, Elahi E, Paisán-Ruiz C. R632W mutation in PLA2G6 segregates with dystonia-parkinsonism in a consanguineous Iranian family. Eur J Neurol. 2009 Jan;16(1):101-4. doi: 10.1111/j.1468-1331.2008.02356.x.
61. Sun YM, Zhou XY, Liang XN, Lin JR, Xu YD, Chen C, et al. The genetic spectrum of a cohort of patients clinically diagnosed as Parkinson's disease in mainland China. NPJ Parkinsons Dis. 2023 May 17;9(1):76. doi: 10.1038/s41531-023-00518-9.
62. Toth-Bencsik R, Balicza P, Varga ET, Lengyel A, Rudas G, Gal A, et al. New Insights of Phospholipase A2 Associated Neurodegeneration Phenotype Based on the Long-Term Follow-Up of a Large Hungarian Family. Front Genet. 2021 Jun 8;12:628904. doi: 10.3389/fgene.2021.628904.
63. Virmani T, Thenganatt MA, Goldman JS, Kubisch C, Greene PE, Alcalay RN. Oculogyric crises induced by levodopa in PLA2G6 parkinsonism-dystonia. Parkinsonism Relat Disord. 2014 Feb;20(2):245-7. doi: 10.1016/j.parkreldis.2013.10.016.
64. Wan Y, Jiang Y, Xie Z, Ling C, Du K, Li R, et al. Novel *PLA2G6*Pathogenic Variants in Chinese Patients With *PLA2G6*-Associated Neurodegeneration. Front Neurol. 2022 Jul 13;13:922528. doi: 10.3389/fneur.2022.922528.
65. Wirth T, Weibel S, Montaut S, Bigaut K, Rudolf G, Chelly J, et al. Severe early-onset impulsive compulsive behavior and psychosis in PLA2G6-related juvenile Parkinson's disease. Parkinsonism Relat Disord. 2017 Aug;41:127-129. doi: 10.1016/j.parkreldis.2017.05.014.
66. Wu MC, Chang YY, Lan MY, Chen YF, Tai CH, Lin YF, et al. A Clinical and Integrated Genetic Study of Isolated and Combined Dystonia in Taiwan. J Mol Diagn. 2022 Mar;24(3):262-273. doi: 10.1016/j.jmoldx.2021.12.003.
67. Xie F, Cen Z, Ouyang Z, Wu S, Xiao J, Luo W. Homozygous p.D331Y mutation in PLA2G6 in two patients with pure autosomal-recessive early-onset parkinsonism: further evidence of a fourth phenotype of PLA2G6-associated neurodegeneration. Parkinsonism Relat Disord. 2015 Apr;21(4):420-2. doi: 10.1016/j.parkreldis.2015.01.012.
68. Yamashita C, Funayama M, Li Y, Yoshino H, Yamada H, Seino Y, et al. Mutation screening of PLA2G6 in Japanese patients with early onset dystonia-parkinsonism. J Neural Transm (Vienna). 2017 Apr;124(4):431-435. doi: 10.1007/s00702-016-1658-7.

69. Yoshino H, Tomiyama H, Tachibana N, Ogaki K, Li Y, Funayama M, et al. Phenotypic spectrum of patients with PLA2G6 mutation and PARK14-linked parkinsonism. Neurology. 2010 Oct 12;75(15):1356-61. doi: 10.1212/WNL.0b013e3181f73649.
70. Zhang P, Gao Z, Jiang Y, Wang J, Zhang F, Wang S, et al. Follow-up study of 25 Chinese children with PLA2G6-associated neurodegeneration. Eur J Neurol. 2013 Feb;20(2):322-30. doi: 10.1111/j.1468-1331.2012.03856.x.
71. Zou Y, Luo H, Yuan H, Xie K, Yang Y, Huang S, et al. Identification of a Novel Nonsense Mutation in *PLA2G6* and Prenatal Diagnosis in a Chinese Family With Infantile Neuroaxonal Dystrophy. Front Neurol. 2022 Jul 6;13:904027. doi: 10.3389/fneur.2022.904027.
